# Evolution of Einkorn wheat centromeres is driven by the mutualistic interplay of two LTR retrotransposons

**DOI:** 10.1186/s13100-024-00326-9

**Published:** 2024-08-05

**Authors:** Matthias Heuberger, Dal-Hoe Koo, Hanin Ibrahim Ahmed, Vijay K. Tiwari, Michael Abrouk, Jesse Poland, Simon G. Krattinger, Thomas Wicker

**Affiliations:** 1 Department of Plant and Microbial Biology, University of Zurich, Zurich, Switzerland; 2 Wheat Genetics Resource Center and Department of Plant Pathology, Kansas State University, Manhattan, KS 66506 USA; 3 Plant Science Program, Biological and Environmental Science and Engineering Division (BESE), King Abdullah University of Science and Technology (KAUST), Thuwal, 23955-6900 Saudi Arabia; 4 Centre d’Anthropobiologie et de Génomique de Toulouse (CAGT), Université Paul Sabatier, Toulouse, France; 5 Department of Plant Science and Landscape Architecture, University of Maryland, College Park, MD 20724 USA

**Keywords:** Centromere evolution, Transposable element population genetics, Centromere stability

## Abstract

**Background:**

Centromere function is highly conserved across eukaryotes, but the underlying centromeric DNA sequences vary dramatically between species. Centromeres often contain a high proportion of repetitive DNA, such as tandem repeats and/or transposable elements (TEs). Einkorn wheat centromeres lack tandem repeat arrays and are instead composed mostly of the two long terminal repeat (LTR) retrotransposon families *RLG_Cereba* and *RLG_Quinta* which specifically insert in centromeres. However, it is poorly understood how these two TE families relate to each other and if and how they contribute to centromere function and evolution.

**Results:**

Based on conservation of diagnostic motifs (LTRs, integrase and primer binding site and polypurine-tract), we propose that *RLG_Cereba* and *RLG_Quinta* are a pair of autonomous and non-autonomous partners, in which the autonomous *RLG_Cereba* contributes all the proteins required for transposition, while the non-autonomous *RLG_Quinta* contributes GAG protein. Phylogenetic analysis of predicted GAG proteins showed that the *RLG_Cereba* lineage was present for at least 100 million years in monocotyledon plants. In contrast, *RLG_Quinta* evolved from *RLG_Cereba* between 28 and 35 million years ago in the common ancestor of oat and wheat. Interestingly, the integrase of *RLG_Cereba* is fused to a so-called CR-domain, which is hypothesized to guide the integrase to the functional centromere. Indeed, ChIP-seq data and TE population analysis show only the youngest subfamilies of *RLG_Cereba* and *RLG_Quinta* are found in the active centromeres. Importantly, the LTRs of *RLG_Quinta* and *RLG_Cereba* are strongly associated with the presence of the centromere-specific CENH3 histone variant. We hypothesize that the LTRs of *RLG_Cereba* and *RLG_Quinta* contribute to wheat centromere integrity by phasing and/or placing CENH3 nucleosomes, thus favoring their persistence in the competitive centromere-niche.

**Conclusion:**

Our data show that *RLG_Cereba* cross-mobilizes the non-autonomous *RLG_Quinta* retrotransposons. New copies of both families are specifically integrated into functional centromeres presumably through direct binding of the integrase CR domain to CENH3 histone variants. The LTRs of newly inserted *RLG_Cereba* and *RLG_Quinta* elements, in turn, recruit and/or phase new CENH3 deposition. This mutualistic interplay between the two TE families and the plant host dynamically maintains wheat centromeres.

**Supplementary Information:**

The online version contains supplementary material available at 10.1186/s13100-024-00326-9.

## Background

In this study, we focus on Einkorn wheat (*Triticum monococcum*), a diploid and the first known wheat species to be domesticated. Einkorn wheat is a member of the Triticeae family which includes important crops such as wheat, barley and rye. The Triticeae belong to the large family of the grasses (Poaceae). Grasses originated probably over 100 million years ago and diversified 50–80 million years ago [45, 65]. The Triticeae diverged from their closest cereal relative, *Avena sativa* (oat) about 28 million years ago and diversified into barley, rye and the wheat group about 10 million years ago [28, 31, 42]. *T. monococcum* itself is a close relative of the A genome of hexaploid (bread) wheat, which diverged about 1 million years ago [28]. Because of their high transposable element (TE) content of > 90%, centromeres from Triticeae had not been sequenced completely [30, 66]. Only very recently, gap-free assemblies of Einkorn wheat centromeres were produced [2], which allowed for the detailed analysis presented in this study.

Centromeres are of fundamental importance for all eukaryotes. During cell division, sister chromatids are moved towards opposing cell poles by microtubules which are connected to chromosomes via the centromeric kinetochore complex. Functional centromeres are defined epigenetically in that the two canonical histone H3 proteins are replaced with histone variants CENH3 (CENP-A in humans) in the nucleosomes [10]. Nucleosomes, which consist of octamers of histone proteins, are placed in regular intervals along chromosomes in a process called “phasing”. Here, DNA stretches of ~ 150 bp are wrapped around each nucleosome with spacers of 10–80 bp separating individual nucleosomes [11].

Centromere sizes vary greatly between species, from “point” centromeres in yeast which are defined by a single ~ 125 bp sequence [14] to *Caenorhabditis elegans* “holo-centromeres” which span nearly the entire length of chromosomes [57]. Plants usually have “regional” centromeres that are several megabases (Mb) in size and typically contain large arrays of centromere-specific tandem repeated sequences [6, 35, 50, 65, 74]. A recent study in the model plant *Arabidopsis thaliana* showed that centromeres contain thousands of tandemly repeated units of the 178 bp sequence motif *Cen178* [35, 73]. Similar tandem repeats were identified in centromeres of several grasses such as rice, maize and *Brachypodium* [6, 65]. Although the centromeric tandem repeat sequences are poorly conserved between species [9], they generally have a size of 150–180 bp, the necessary length for DNA to wrap around one nucleosome and allowing for spacing between nucleosomes.

In most grasses studied so far, centromeric tandem repeats are interspersed with LTR retrotransposons from highly centromere-specific families [6, 50, 65, 74]. The known centromere-specific retrotransposon families in grasses all belong to the *Gypsy* superfamily, and sequence homology indicates that they all evolved from a common ancestor in flowering plants (angiosperms, [38]). Occasionally, they may have been transferred horizontally between species [55]. In maize and rice, they are referred to as *CRM* (centromeric retrotransposons of maize) and *CRR* (centromeric retrotransposons of rice), respectively [39, 54], while in Triticeae they are called *RLG_Cereba* [46, 69]. Interestingly, a recent study found that Einkorn wheat does not have centromere-specific tandem repeats. Instead, its highly repetitive centromeres are derived almost exclusively from retrotransposons, with the *RLG_Cereba* and *RLG_Quinta* families being the most abundant [2].

Despite the low sequence conservation of centromere-specific repeats between species, the recruitment and/or phasing of centromeric (CENH3 containing) nucleosomes is likely promoted by specific DNA sequence motifs [9]. In yeast, for example, a specific ~ 125 bp sequence is essential for the establishment of its point centromeres [8]. In Arabidopsis, the *Cen178* tandem repeats are strongly associated with CENH3 histone variants, while interspersed TEs in centromeres show much lower CENH3 signals [35]. Additionally, more divergent *Cen178* copies were less associated with CENH3, suggesting selection pressure for specific sequence motifs [35]. In grasses, the DNA of some centromere-specific retrotransposons was shown to interact with CENH3 and their transcription might be involved in CENH3 deposition [75, 18]. In particular, parts of *RLG_Cereba* and *RLG_Quinta* retrotransposons from hexaploid wheat were shown to have strong affinity for CENH3 [22, 27, 58]. Interestingly, even the human immunodeficiency virus (HIV) was shown to have strictly placed nucleosomes in its LTR which are important for the transcriptional regulation of the retrovirus [36].

It has long been known that some retrotransposons target epigenetic marks for insertion into the genome, and that this is promoted by specific chromodomains that are fused to the C-terminus of the integrase (INT) proteins [1, 12]. Centromere-specific retrotransposons from grasses lack the classic chromodomain but instead contain a so-called CR domain [12, 37, 38]. It was therefore hypothesized that the CR domain recognizes functional centromeres and guides new insertions there. However, the exact function of the CR domain and the molecular mechanism underlying the centromere targeting are still unknown. Additionally, there may be other mechanisms that lead to accumulation of TEs in centromeric and pericentromeric regions. For example, the *Athila* retrotransposons in Arabidopsis are prevalent in peri/centromeric regions but do not contain CR domains [37, 43, 44]. It is unclear whether they actively target centromeres or whether they simply accumulate where they have the least deleterious effects.

Here, we analyze recently published gap-free assemblies of *T. monococcum* (Einkorn wheat) centromeres. We show that the centromere-specific *RLG_Quinta* elements are non-autonomous and most likely cross-mobilized by *RLG_Cereba* elements, and that new *RLG_Quinta* and *RLG_Cereba* insertions indeed target functional centromeres. Additionally, using ChIP-seq data, we identified specific sequences inside *RLG_Quinta* and *RLG_Cereba* LTRs which strongly phase CENH3-containing nucleosomes. Our combined findings allow the development of a model for the dynamic evolution of centromeres in wheat that is driven by the *RLG_Quinta* and *RLG_Cereba* retrotransposon families.

## Results and discussion

### *RLG_Cereba* is an autonomous retrotransposon that cross-mobilizes *RLG_Quinta*

For our previous study, we identified the boundaries of functional centromeres in Einkorn wheat accessions TA299 and TA10622 with the help of CENH3 ChIP-seq data [2]. For the current study, we focused on TA299 because its centromeres were assembled gap-free, while the centromere of chromosome 2A in the TA10622 assembly still contains a few gaps. We re-annotated centromeres with a specific focus on centromeric retrotransposons using an in-house pipeline. The known centromere-specific *RLG_Cereba* and *RLG_Quinta* retrotransposon families contribute ~ 47% and ~ 9% of the functional centromeric sequences, while they are practically absent from chromosome arms (Supplementary Fig. S1). The third known centromere-specific family, *RLG_Abia* [69], contributes less than 5%. Other high-copy TE families are also present, but far less abundant than outside centromeres (Supplementary Fig. S1).

Using recently described methods [71], we isolated 2,658 full-length *RLG_Cereba* and 935 *RLG_Quinta* retrotransposon copies from the TA299 *T. monococcum* assembly. From these we derived consensus sequences for multiple subfamilies to predict open reading frames (ORFs), encoded proteins and structural motifs such as primer binding sites (PBS) or poly-purine tracts (PPT). Analogous analyses for accession TA10622 yielded practically identical consensus sequences.

**Fig. 1:**
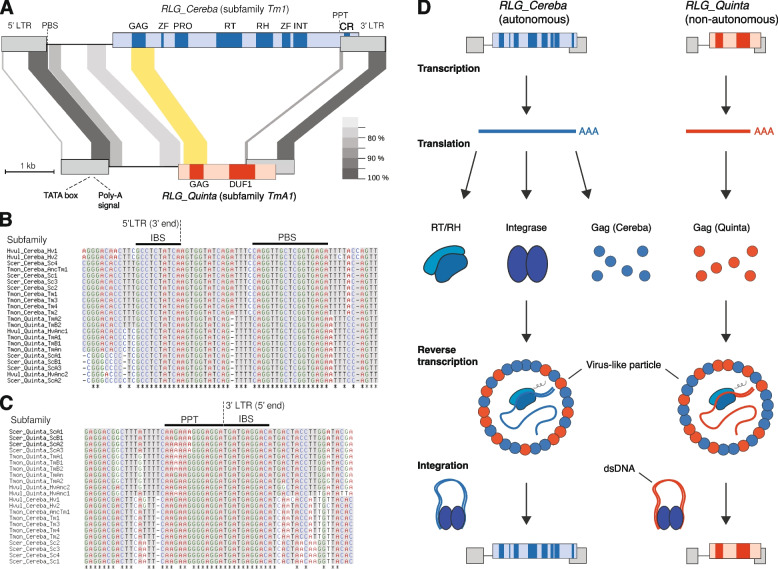
Characterisation of centromere-specific *RLG_Cereba* and *RLG_Quinta* retrotransposons. **A** Comparison of sequence organization of *RLG_Cereba* and *RLG_Quinta*. Sequences conserved at the DNA level are connected with gray areas, with the shade of gray reflecting the level of sequence conservation. The region encoding the GAG domain, which shows conservation at the protein but barely any homology at the DNA level, is indicated by a yellow area. *RLG_Cereba* encodes a polyprotein with typical domains for autonomous retrotransposons, plus a previously described CR motif. In contrast, *RLG_Quinta* encodes only a GAG domain and a domain of unknown function (DUF1). Note that parts of the LTRs including diagnostic motifs such as LTR termini and primer binding site (PBS) are highly conserved between the two. ZF: Zinc finger, PRO: aspartic protease, RT: reverse transcriptase, RH: RNase H, INT: integrase, PPT: poly-purine tract. **B** Multiple sequence alignment of 3’ terminal regions of 5’ LTRs from *RLG_Cereba* and *RLG_Quinta* subfamilies. Predicted integrase binding site (IBS) and PBS are indicated with black horizontal bars. **C** Multiple sequence alignment of 5’ terminal regions of 3’ LTRs from *RLG_Cereba* and *RLG_Quinta* subfamilies. Predicted PPT and IBS are indicated with black horizontal bars. **D** Schematic model of how *RLG_Quinta* is cross-mobilized by *RLG_Cereba*. Both TEs are transcribed at the same time due to conservation of regulatory regions. *RLG_Cereba* provides proteins necessary for replication (e.g. RT and INT), while *RLG_Quinta* contributes GAG proteins which are needed in large quantities for the formation of virus-like particles

*RLG_Cereba* contains one large ORF that encodes all canonical proteins necessary for its replication, namely GAG that forms virus-like particles in which reverse transcription takes place, reverse transcriptase (RT), RNAse H (RH), integrase (INT) and a protease that cleaves the polyprotein into its functional enzymes (Fig. 1).

Additionally, the INT protein has the CR domain that is exclusively found in centromere-specific retrotransposons [37] (see below). In contrast, *RLG_Quinta* retrotransposons are shorter and contain an ORF that only encodes GAG and a domain of unknown function (DUF1, Fig. 1a), while it lacks coding sequences (CDS) for RT and INT. While homology to GAG is very clear, at this point we have no hint as to the function of DUF1. Thus, we consider *RLG_Quinta* a non-autonomous retrotransposon that must rely on enzymes encoded elsewhere for its replication. However, the intact ORF for GAG indicates that it contributes GAG protein which can be used by both *RLG_Cereba* and *RLG_Quinta*.

It is not unusual that TE populations in plants comprise autonomous and non-autonomous elements [70], and several of the most abundant TEs in Triticeae were shown to be non-autonomous [70, 71]. Indeed, *RLG_Cereba* and *RLG_Quinta* fulfill the previously described criteria [71] for a pair of autonomous and non-autonomous retrotransposons (Fig. 1): first, the predicted primer binding site (PBS) just downstream of the 5’ LTR which is needed for the initiation of reverse transcription is identical in both families (Fig. 1B). Second, the termini of the LTRs, which serve as binding sites of the integrase protein are identical in all identified *RLG_Cereba* and *RLG_Quinta* subfamilies from Triticeae (Fig. 1B and C). Third, the second half of the LTR (which contains putative promoter and terminator sequences) is strongly conserved between *RLG_Cereba* and *RLG_Quinta* elements (Fig. 1A, Supplementary Fig. S2). The conserved 3’ half of the *RLG_Cereba* and *RLG_Quinta* LTR starts near a predicted TATA box (Fig. 1, Supplementary Fig. S2). The conserved region also contains a putative poly-adenylation signal which we identified with the help of published IsoSeq transcripts for both families [2]. *RLG_Cereba* has the canonical AATAAA poly-adenylation signal ~ 12 bp upstream of the start of the poly-A tail in the IsoSeq transcript (Supplementary Fig. S2B), while *RLG_Quinta* has two cryptic poly-adenylation sequences (AATA and ATATATAT) approximately 20–30 bp upstream of the poly-A tail. Importantly, the predicted TATA box and poly-adenylation signal are both supported by homology to the promoter of HIV. Here, the known TATA box [4] as well as the AATAAA motifs plus surrounding sequences are conserved between HIV and the two retrotransposons (Supplementary Fig. S2C). Based on this analysis, we inferred the boundaries of the typical U3, R and U5 regions of the *RLG_Cereba* and *RLG_Quinta* LTRs (Supplementary Fig. S2C). The conservation of the regulatory motifs in the 3’ half of their LTRs suggest that *RLG_Cereba* and *RLG_Quinta* may be co-expressed at the same time.

Finally, *RLG_Cereba* is the only retrotransposon family in the extensive datasets of wheat TEs that shares the described features with *RLG_Quinta*. We therefore conclude that *RLG_Cereba* is the autonomous partner that mobilizes *RLG_Quinta* elements, while *RLG_Quinta* contributes GAG protein. The latter makes *RLG_Quinta* a partially mutualistic partner in the *RLG_Cereba*/*RLG_Quinta* system, since GAG proteins are needed in large quantities for the formation of virus-like particles in which replication takes place. This is reminiscent of the “semi-autonomous” *RLG_Sabrina* retrotransposons in wheat which also sometimes encode GAG but lack genes for RT and INT [71].

### *RLG_Quinta* evolved from *RLG_Cereba* in a common ancestor of oat and wheat

**Fig. 2:**
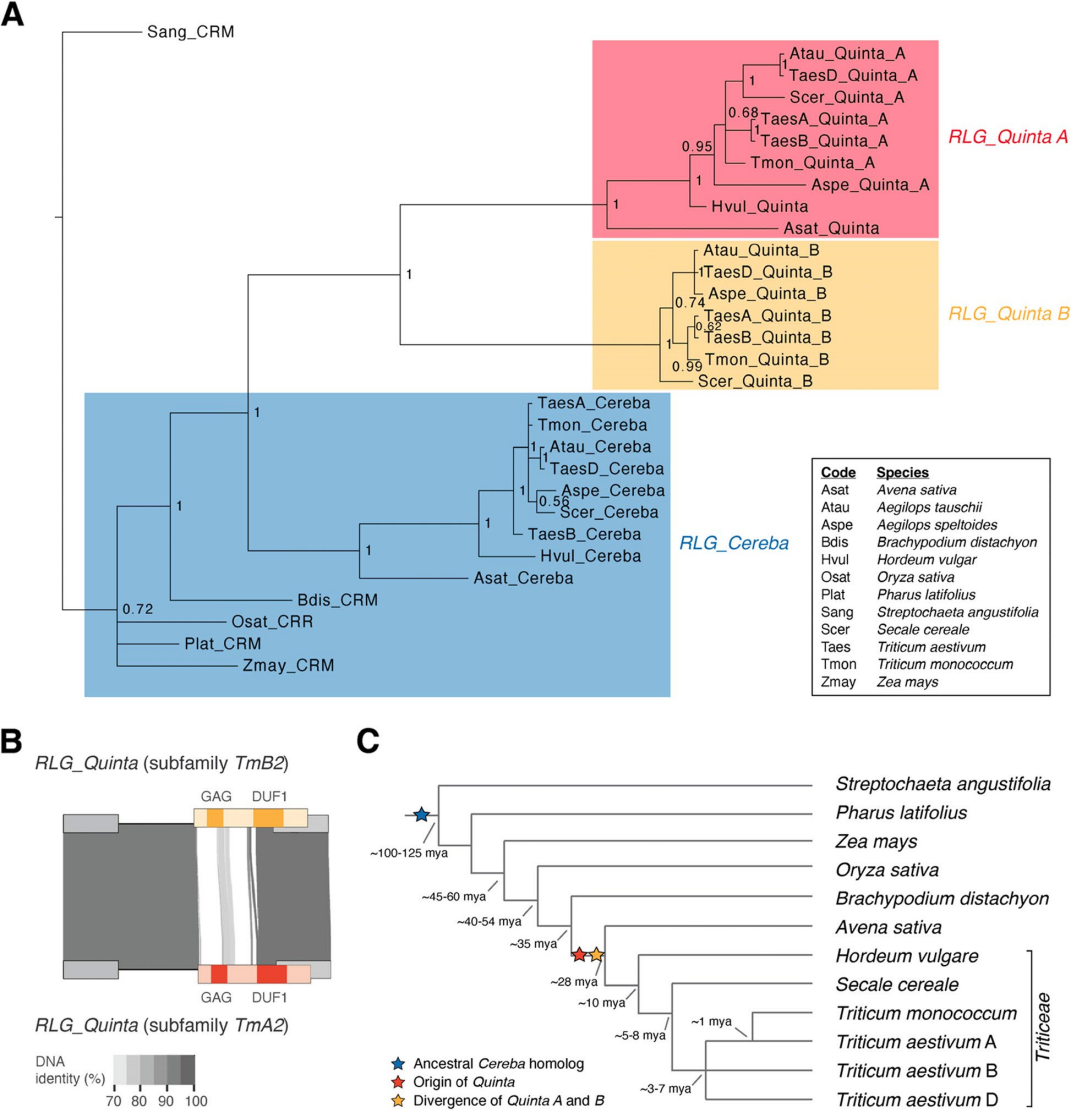
Evolutionary origin of *RLG_Cereba* and *RLG_Quinta* retrotransposons. **A** Phylogenetic tree of predicted GAG proteins from *RLG_Quinta, RLG_Cereba* and homologs from other grasses. The tree was constructed with MrBayes running for 125,000 generations. The numbers at branches indicate the probability that the taxa to the right of the branch are grouped together in all trees. The tree shows that *RLG_Quinta* evolved in the evolutionary lineage leading to Triticeae and oat (*A. sativa*). *RLG_Quinta* subsequently diverged into two main branches (A and B). **B** Sequence comparison of the two main *RLG_Quinta* lineages A and B using consensus sequences for two representative subfamilies from *T. monococcum*. The region of the GAG coding sequence shows very low sequence conservation at the DNA level, while the remaining sequence can be well aligned. This indicates that *RLG_Quinta* lineages underwent multiple recombination events involving GAG CDS and flanking sequences (see also Supplementary Fig. S3). **C** Schematic phylogeny of the grasses (Poaceae). Divergence times in million years are shown at separation nodes. Divergence times are approximate and were compiled from multiple publications (see text). Phylogenetic emergence of *RLG_Cereba* and *RLG_Quinta* retrotransposon lineages is indicated with stars

The finding that *RLG_Quinta* is a non-autonomous derivative of *RLG_Cereba* raised the question when *RLG_Cereba* and *RLG_Quinta* elements first evolved. For phylogenetic and comparative analysis, we isolated *RLG_Cereba* homologs from Triticeae (barley, rye, wheat) and their close relative oat (*A. sativa*). Additionally, we searched more distantly related grasses, *Brachypodium*, rice and maize, as well as the basal grasses *Pharus latifolius* and *Streptochaeta angustifolium*. We found *RLG_Cereba* homologs in all species studied, which dates the origin of *RLG_Cereba* homologs at least to a common ancestor of *Streptochaeta* and wheat, or approximately 100 million years ago [45], Fig. 2). In fact, *RLG_Cereba* homologs have been described in a wide range of plants including dicotyledons, indicating that they are an ancient retrotransposons lineage [38].

In contrast to *RLG_Cereba*, we identified *RLG_Quinta* homologs only in Triticeae and oat, dating their emergence to the period between 35 and 28 MYA, after the Triticeae/oat ancestor diverged from *Brachypodium* (Fig. 2). This is also reflected in the phylogenetic tree of predicted GAG proteins where the *RLG_Quinta* lineage branched off after the divergence of Triticeae and oat from the other grasses (with strong branch support of 100%, Fig. 2A). We therefore conclude that *RLG_Quinta* evolved from a loss of the CDS for RT and INT, while the CDS for GAG was still maintained. The additional domain of unknown function encoded by *RLG_Quinta* may have been acquired later. Adding complexity, *RLG_Quinta* elements diverged early on into two main lineages (A and B) which encode two variants of GAG proteins that strongly differ from each other and from GAG encoded by *RLG_Cereba* (Fig. 2A and B). Interestingly, *RLG_Quinta_A* and *RLG_Quinta_B* also have highly divergent GAG genes where DNA identity is only ~ 65% (Fig. 2B, Supplementary Fig. S3), while sequences up- and downstream of the GAG genes are over 90% identical (Fig. 2B). This indicates that the *RLG_Quinta* A and B lineages recombined during their evolution (Supplementary Fig. S3). Such sequence exchange is well studied in retrotransposons and retroviruses and likely occurs through template switching during replication [15, 63]. We hypothesize that *RLG_Quinta* contributes with specific GAG variants to the *RLG_Cereba/RLG_Quinta* system, similar to previously described non-autonomous *RLG_Sabrina* and *RLG_WHAM* retrotransposons in wheat (Fig. 1D, [71]).

The phylogenetic tree of GAG proteins largely reflects the phylogeny of the Triticeae and their relatives, indicating no horizontal transfer between main taxa, at least since Triticeae diverged from the *Brachypodium* lineage (Fig. 2A).

### Diversification of retrotransposon subfamilies during recent evolution

Using previously described methods [71], we performed principal component analysis (PCA) of populations of *RLG_Cereba* and *RLG_Quinta* elements. Here, we aligned all individual full-length *RLG_Cereba* and *RLG_Quinta* copies to their respective consensus sequences. From these alignments, sequence variants were called that were then used for PCA. This analysis was done with retrotransposon copies from seven genomes. This included rye (*S. cereale*), the three subgenomes of hexaploid wheat (A, B and D genome) as well as their diploid relatives *T. monococcum*, *Aegilops speltoides* and *Aegilops tauschii*, respectively. Barley was excluded since its centromeres are still assembled incompletely and do not contain sufficient numbers of full-length retrotransposon copies [30].

**Fig. 3:**
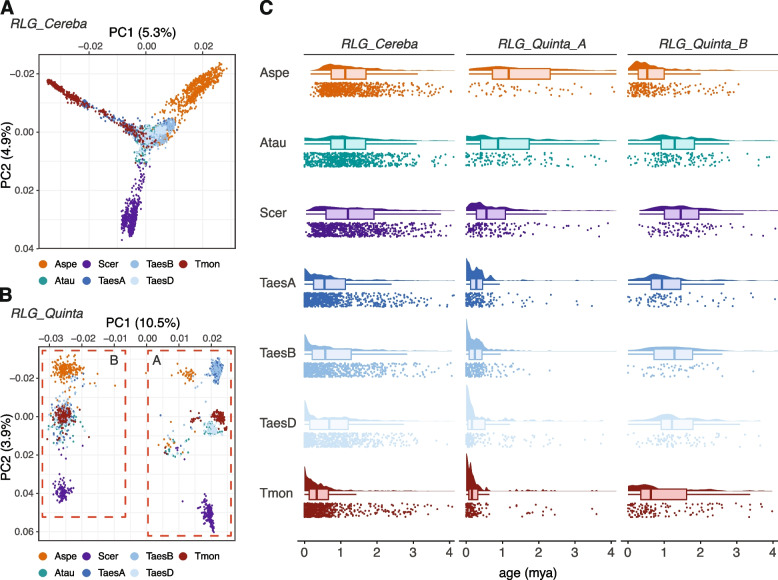
Analysis of populations of centromere-specific retrotransposons. **A** Principal component analysis (PCA) of full length *RLG_Cereba* elements using SNPs obtained from alignment of individual elements against a consensus sequence. For each species, 747 randomly picked elements were included in the analysis. Retrotransposons form separate groups which largely correspond to the species/subgenomes used (Aspe: *Ae. speltoides*, Atau: *Ae. tauschii*, Scer: *S. cereale*, Tmon: *T. monococcum* (accession TA299), Taes: *T. aestivum*, A, B and D subgenomes). **B** The same analysis as in (**A**) but using 274 randomly picked *RLG_Quinta* elements from each species/subgenome. A and B clusters include elements from all the included species. **C** Insertion age distributions estimated from LTR divergence of *RLG_Cereba* and *RLG_Quinta* elements. Colors correspond to those of the subfamilies shown in the PCA in **A** and **B**. For visual clarity only data points falling into the 99% percentile are shown. The most recently active *RLG_Quinta* elements in the *T. aestivum* subgenomes and *T. monococcum* are part of the A lineage, while the B lineage was more recently active in the genome of *Ae. speltoides*

For *RLG_Cereba*, the PCA largely reflects species phylogeny (Fig. 3A, Supplementary Fig. S4A for accession TA10622), with rye, *Ae. speltoides* and *T. monococcum* diverging from the core of the wheat subgenomes. The A, B and D subgenomes are in the center of the PCA possibly because the consensus sequence was done from randomly picked copies from all species, of which wheat subgenomes provide the majority. In any case, the PCA shows that distinct subfamilies diverged in the different species and subgenomes since they evolved from a common ancestor 3–6 million years ago [28, 31].

In contrast, *RLG_Quinta* retrotransposon populations are more diverse than those of *RLG_Cereba.* The first principal component (PC1) separates the two main lineages A and B which were already identified in the phylogenetic tree (Fig. 2B) and which were present in the Triticeae/oat ancestor. The clear separation is due to strong divergence in the region encoding the GAG protein domain where sequences between the A and B lineage can hardly be aligned (Fig. 2B). The second principal component (PC2) separates subfamilies mainly reflecting the different species (Fig. 3B, Supplementary Fig. S4B for accession TA10622).

In all species studied here, most *RLG_Cereba* and *RLG_Quinta* copies inserted in the past 4 million years. This is typical for TEs in grasses, because intergenic sequences are rapidly reshuffled through TE insertions and deletion of DNA [64], making it rare to find TEs older than a few million years. Interestingly, especially *T. monococcum* centromeres contain large numbers of very young *RLG_Cereba* and *RLG_Quinta* copies. Here, the *RLG_Quinta_A* lineage was more recently active, with most copies being less than 200,000 years old (Fig. 3C, Supplementary Fig. S4C). This could in part be due to the high quality of the assembly but could also reflect very recent insertion activity (see below).

### *RLG_Cereba* and *RLG_Quinta* insert specifically into functional centromeres

It was proposed that the CR domain fused to the INT protein of centromere-specific retrotransposons guides their insertion to functional centromeres [37, 38]. Indeed, previous studies in wheat found that the younger *RLG_Cereba* elements tend to be in the middle of centromeres [71]. However, sequence assemblies in centromeric regions were not complete enough to answer the question whether they indeed actively target functional centromeres, or whether they are simply tolerated best in centromeric regions.

**Fig. 4:**
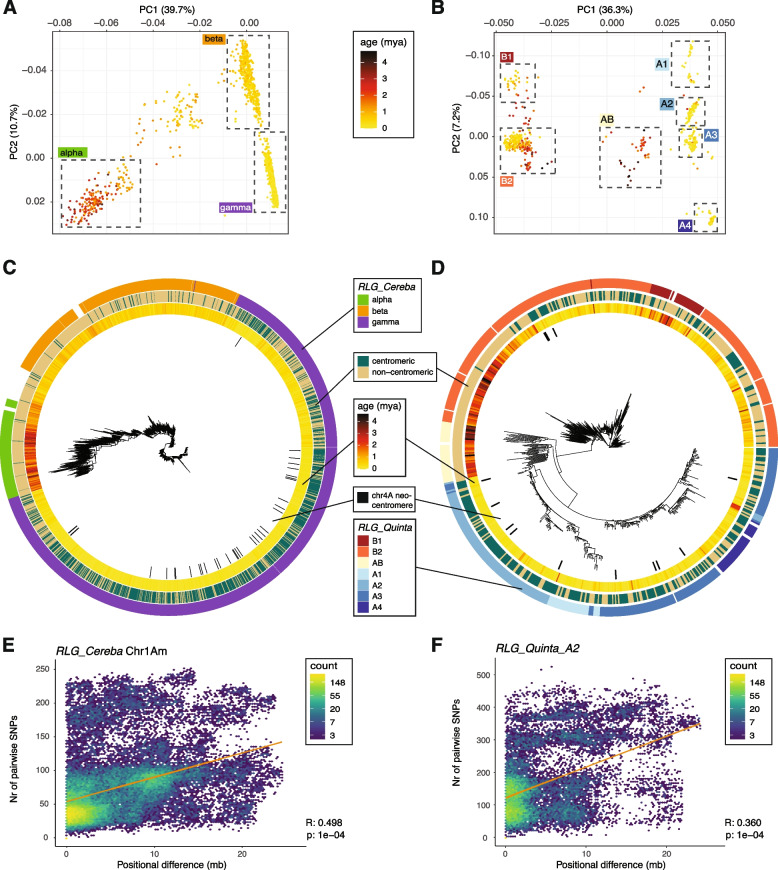
Analysis of insertion age and physical location of *RLG_Cereba* and *RLG_Quinta* retrotransposons from *T. monococcum *(accession TA299). **A** Principal component analysis (PCA) of 1,964 full length *RLG_Cereba* elements using SNPs obtained from alignment of individual copies against a consensus sequence. **B** The same analysis with 663 *RLG_Quinta* copies. **C** and **D** Phylogenetic trees for individual *RLG_Cereba* and *RLG_Quinta* copies inferred by RAxML. **E** and **F** Association of physical to genetic distance of retrotransposon copies in centromeres. The x-axis indicates the difference in genomic position, measured by the absolute difference in the distance from the centromere midpoint. The y-axis shows the number of SNPs in pairwise alignments of individual copies. Mantel test statistics and the corresponding *p*-value are shown in the bottom right of each plot. The orange line shows the linear regression calculated by the function lm(). Other chromosomes for *RLG_Cereba* and *RLG_Quinta_B* are shown in Supp. Fig. S5 and S6

Here, we analyzed sequence diversity, phylogeny, insertion age and positions of 1,964 *RLG_Cereba* and 663* RLG_Quinta* elements from *T. monococcum.* To exclude possible recombinant copies, we only used copies which had target site duplications (TSDs) with maximum 1 bp mismatch. By PCA, we distinguished 3 subfamilies for *RLG_Cereba* and 7 for *RLG_Quinta*, which were also reflected in their phylogenetic trees (Fig. 4). The phylogenetic trees show that the different subfamilies were active at different times during the past 4 million years. Older copies are mostly found outside of functional centromeres, while younger ones are inside (Fig. 4). Moreover, in our recent study, we found that the centromere of chromosome 4 has shifted 20,00–100,000 years ago by approximately 10 Mb [2]. This functional neocentromere contains the youngest insertions of *RLG_Cereba* and *RLG_Quinta* elements, indicating that active centromeres are indeed targets for new insertions.

We performed the Mantel test which is commonly used in population genetics to find correlations between genetic and geographical distances. We tested whether we find a correlation between sequence similarity between individual copies and their physical distance to the centromere. Here, we calculated the distance from the centromere midpoint for each TE copy. The positional difference between two copies was then defined as the absolute difference between their distances from the centromere midpoint. For example, if the centromere midpoint for a chromosome is at position 200 Mb, and two copies are located at 180 Mb and 210 Mb on this chromosome, their positional difference would be 10 Mb. Indeed, we found a strong correlation between genetic and physical distance for both *RLG_Cereba* and *RLG_Quinta* elements (Fig. 4E and F, Supplementary Fig. S5 and S6). This indicates that copies that were active at the same time were also inserted into similar physical loci (i.e. the functional centromeres), while older copies were “pushed” away from the centromeres over time. This is in line with the previous observation that the youngest *RLG_Cereba* elements are found almost exclusively in centromeres [2, 66].

Taken together, our analyses provide strong evidence that the *RLG_Cereba* integrase indeed actively targets functional centromeres.

### The CR domain likely guides retrotransposon insertions toward functional centromeres

**Fig. 5:**
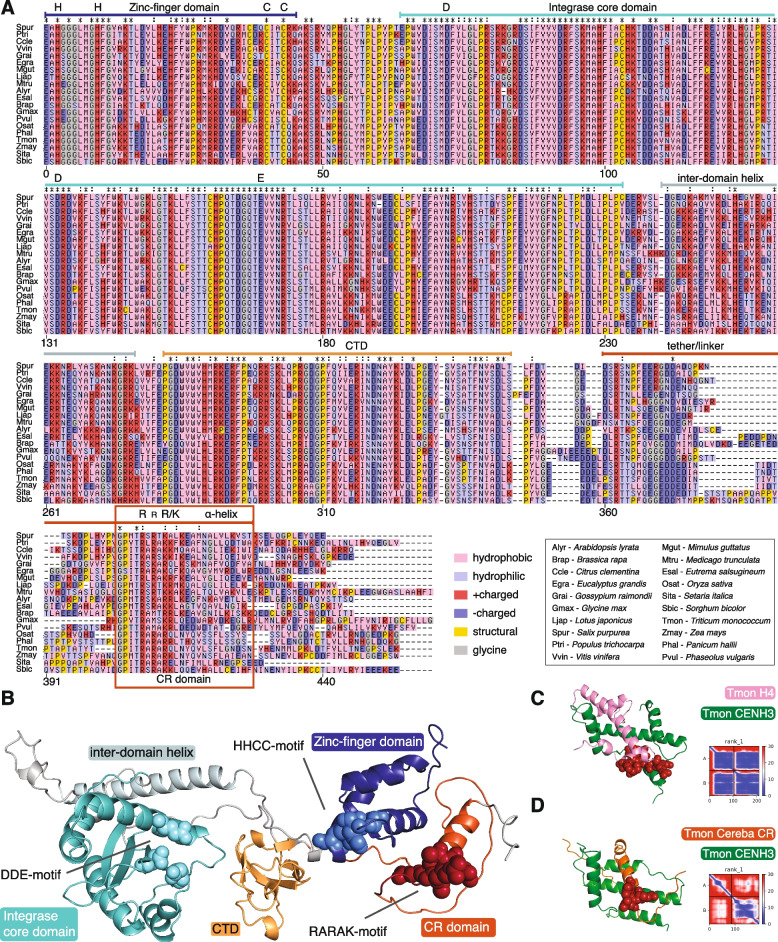
Analysis of integrase sequences from *RLG_Cereba* homologs. **A** Multiple alignment of predicted integrase proteins containing CR domains from 20 plant species. The previously described protein domains are indicated with colored bars above the aligned sequences. Diagnostic residues of the zinc finger, the DDE catalytic site and CR domain are shown. **B** Alphafold2 model of the *RLG_Cereba* integrase. Protein domains are indicated with the same colors as in (A) and diagnostic residues are shown as spheres. **C** Alphafold2 model for the interaction of histone H4 with CENH3 from *T. monococcum* (Tmon). Positively charged amino acids that interact with the negatively charged back bone of the DNA are shown as spheres. CENH3 interacts with H4 in the same way as human CENP-A (see Supplementary Fig. S7). The inset shows the predicted aligned error (PAE) plot. **D** The predicted interaction of the CR domain with CENH3. Note that the alpha helix and the RARAK motif (spheres) interact in a similar way with CENH3 as H4 (see also Supplementary Fig. S7)

In wheat, *RLG_Cereba* and the much less abundant *RLG_Abia* are the only autonomous LTR retrotransposons strongly enriched in centromeric and peri-centromeric regions [69, 71], Supplementary Fig. S1). They are also the only TE families in the extensive wheat TE datasets which contain a CR domain fused to their INT protein (Fig. 5). A previous study on *Gypsy* superfamily retrotransposons showed that CRM homologs (which includes *RLG_Cereba*) formed a monophyletic clade, indicating that the acquisition of the CR domain was a one-time evolutionary event [38].

We hypothesized that the centromere-specificity is caused by a direct interaction of the CR domain with the centromeric CENH3 histone variants. We therefore compared integrase domains from centrome-specific retrotransposons from 20 plant species, covering the two major plant clades of the Monocotyledons and Dicotyledons. In all analyzed sequences, the integrase is well conserved, containing the typical domains, the HHCC Zinc finger, the integrase core domain which contains the catalytic DDE motif and a C-terminal domain (CTD, Fig. 5B). The CR domain itself is characterized by an alpha helix at which start lies a conserved TRARAR/K motif (hereafter RARAK, Fig. 5). It is separated from the canonical integrase by a poorly conserved “spacer” or “tether”.

We then used Alphafold2 to model possible interactions between the CR domain and CENH3-containing nucleosomes. The structure of human centromeric nucleosomes is well known [59], Supplementary Fig. S7) and shows direct interaction of CENP-A (the human CENH3 homolog) with histone H4. As a control, we modeled the interaction of CENH3 and histone H4 from *T. monococcum*, which looked practically identical with the dimer of the two human proteins (Fig. 7C). Interestingly, replacing H4 with the CR domain of *RLG_Cereba* resulted in a very similar dimer in which, most notably, the positively charged R and K residues of the RARAK motif were in the same position as R and K residues in H4, near the negatively charged back bone of the DNA wrapping around the nucleosome (Fig. 5D, Supplementary Fig. S7).

We are aware of the uncertainty of predictions of protein–protein interactions. Nevertheless, our results suggest that the CR domain may interact with CENH3 in a manner similar to H4. It is, for example, possible that the CR domain competes with H4 when nucleosomes are assembled during DNA replication [53]. This would “anchor” the integrase/dsDNA complex and guide the insertion to a nearby location, for example to one of the neighboring coils of the chromatin fiber (see below).

### The presence of CENH3 is strongly associated with *RLG_Cereba* and *RLG_Quinta* LTRs

If *RLG_Cereba* INT indeed guides insertions toward the functional centromere, it would explain why other centromeric repeats would over time be out-competed. We therefore hypothesized that *RLG_Cereba* and/or *RLG_Quinta* should also functionally replace sequences where CENH3 containing nucleosomes are positioned. For our previous study, we produced ChIP-seq data to localize the boundaries of functional centromeres [2]. The main ChIP-seq experiment used MNase digested DNA and enrichment for CENH3 by antibody, while the H3K4me3 histone modification (which characterizes open chromatin) was used as control. As negative controls, ChIP-seq sequencing was done with MNase digested DNA without the use of antibodies. In this study, we used the ChIP-seq data to search for sequence motifs which promote deposition and/or phasing of CENH3 containing nucleosomes. We mapped CENH3 ChIP-seq reads on *T. monococcum* chromosomes, allowing multi-mapping reads to include regions that are highly conserved within TE families (see methods). To minimize erroneous mappings, we only allowed perfect alignment matches of at least 150 bp.

**Fig. 6:**
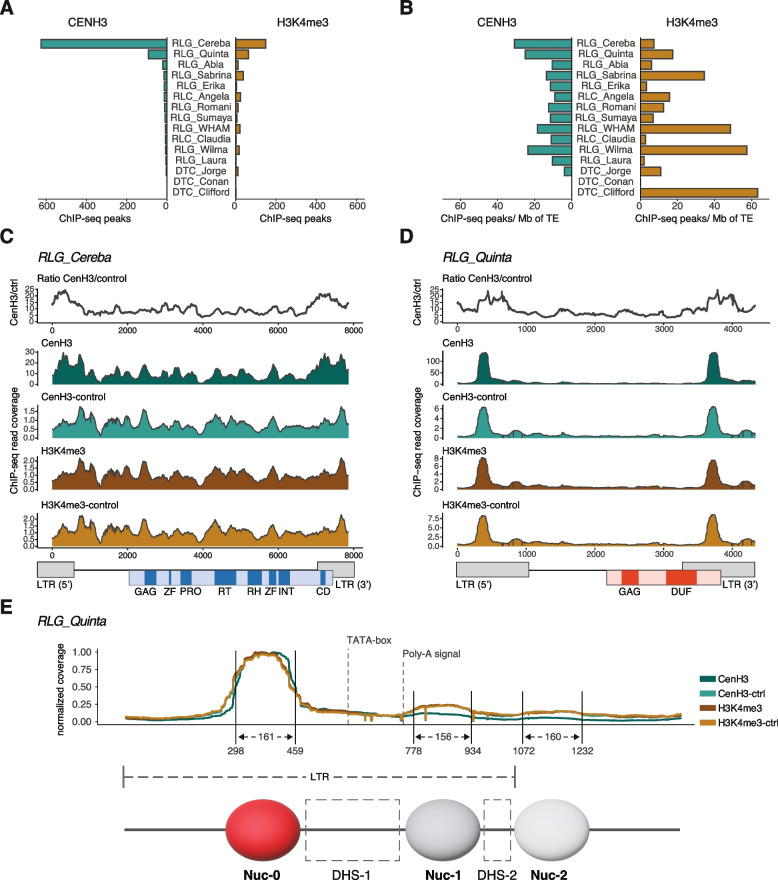
Analysis of ChIP-seq data in centromeric TEs from *T. monococcum*. **A** Numbers of ChIP-seq peaks found in TE sequences. A ChIP-seq peak was defined as a region of 80–300 bp which showed high read coverage in the experimental set (i.e. CENH3 and H3K4me3) but no signal in controls (see methods). **B** The same data normalized to the abundance and size of TE families. **C** and **D** ChIP-seq read coverage mapped on *RLG_Quinta* and *RLG_Cereba* consensus sequences. Here, ChIP-seq read coverage of all available full-length copies was compiled. The individual tracks show read coverage from different experiments and mock controls (MNase digested DNA without the use of antibodies). The top track shows the ratio between CENH3 and its mock inoculation. **E** Detailed mapping of the terminal 2000 bp of *RLG_Quinta*. ChIP-seq read coverage was used to infer positioning of nucleosomes. Nucleosomes and DNAse hypersensitive regions (DHS) were named analogous to those in human immunodeficiency virus (HIV [36])

We first aimed at identifying loci which showed a high coverage with CENH3 ChIP-seq reads but low coverage in control experiments (see methods). In this “sequence agnostic” approach, we identified 1,051 sequences with an average size of 146 bp inside functional centromeres. A total of 933 (~ 89%) of them had homology to TEs. For the H3K4me3 control, we identified 675 loci, of which 495 (~ 73%) were TE-derived (Fig. 6). This indicates that in wheat centromeres, only a fraction of nucleosomes contains CENH3. It can also be expected that CENH3 localization varies somewhat between individual cells, or that individual nucleosomes are heterotypic (i.e. they may contain one H3 and one CENH3 histone variant). With our method, we selected those loci which were consistently associated with CENH3 while having very low signals in control data.

We mapped the 1,051 CENH3-associated sequences on wheat TE consensus sequences and found that they are highly enriched in *RLG_Cereba* and *RLG_Quinta* sequences, while other TE families showed only very low coverage (Fig. 6). In contrast, the 675 sequences found associated with H3K4me3 were enriched in various TE families other than *RLG_Cereba* and *RLG_Quinta* (Fig. 6A and B). Normalization for TE abundance shows that TE families other than *RLG_Cereba* and *RLG_Quinta* are associated more often with the H3K4me3 control (Fig. 6B), while *RLG_Cereba* and *RLG_Quinta* are under-represented. Taken together, these data indicate that a large majority of CENH3 signals in centromeres are associated with *RLG_Cereba* and *RLG_Quinta,* and that the two families have a particular affinity to CENH3 containing nucleosomes. Similar observations were made in hexaploid wheat, where both families showed co-localization with CENH3 and ChIP enrichment [22, 27]. Outside centromeres, we found no particular enrichment of TE families in either experiment (Suppl Fig. S8). Furthermore, the CENH3 phasing satellite repeats T566 and T550 previously described in hexaploid wheat [58] were found only in few copies and predominantly outside of centromeres, reflecting the previous finding that *T. monococcum* has lost practically all centromeric tandem repeats [2].

Since CENH3 reads were enriched for *RLG_Cereba* and *RLG_Quinta* sequences, we cross-matched sequence read coverage of all ChIP-seq experiments with our annotation of full-length TEs. This was then used to map read coverage on consensus sequences of the two families allowing examination of read coverage at a near base pair resolution. Interestingly, CENH3-associated sequences came particularly often from *RLG_Cereba* and *RLG_Quinta* LTRs (Fig. 6C and D). Additionally, the ratio between CENH3 and CENH3 mock was highest in LTRs (Fig. 6C). These data indicate that *RLG_Cereba* and *RLG_Quinta* LTRs have a particular affinity for CENH3 containing nucleosomes (Fig. 6C and D).

### A 162 bp motif in the *RLG_Quinta* LTR is associated with strict placement of nucleosomes

MNase treatment is known to be very sensitive to over-digestion of DNA [51] and some levels of over-digestion have to be expected. This can lead to a general enrichment of sequence motifs that are (i) MNase resistant and (ii) strongly phasing nucleosomes (i.e. strictly keeping nucleosomes in place once they associate with the given sequence). In our case, this led to the identification of a particular ~ 162 bp motif in the 5’ half of the *RLG_Quinta LTR* (the region which is not conserved between *RLG_Cereba* and *RLG_Quinta* LTRs, Supplementary Fig. S9A, see Fig. 1A). This sequence (hereafter called *QuinCent*) showed 10–15 times the average read coverage in all samples, including controls and mock inoculations (Fig. 6D). This indicates that the *QuinCent* motif generally interacts with nucleosomes and holds them strictly in place. It also suggests that this sequence is highly resistant to MNase treatment, which would explain its high abundance in the two mock controls. The *QuinCent* motif has a cryptic inverted repeat structure over most of its length (Suppl. Fig. S9B). We speculate that this could be a functional feature, as it could make binding of nucleosomes strand independent. There are various studies on conserved sequence motifs which promote nucleosome positioning (e.g. [24, 60, 61]). However, we did not find any of the previously described motifs. It is thus possible that the *QuinCent* sequence represents a novel type of nucleosome positioning sequence.

A strict positioning/phasing of nucleosomes should, consequently, influence the placements of neighboring nucleosomes. This is indeed visible in our mapping of ChIP-seq coverage on the *RLG_Quinta* consensus sequence (Fig. 6D and E): the *QuinCent* motif presumably acts as an “anchor” point which determines the placement of nearby nucleosomes, which is visible in weaker but periodic neighboring peaks in ChIP-seq read coverage (Fig. 6D and E. The proposed placing/phasing of CENH3 containing nucleosomes in the LTR of *RLG_Quinta* is surprisingly similar to the situation described for the LTR of HIV where nucleosomes Nuc-0, Nuc-1 and Nuc-2 are strictly placed in the LTR [36], defining a region accessible to transcription factors between Nuc-0 and Nuc-1. The proposed nucleosome positioning in *RLG_Quinta* places Nuc-0 and Nuc-1 up- and downstream of the region that shows homology to the HIV promoter, and which contains the predicted TATA box (Fig. 6E).

Because the Nuc-0 binding *QuinCent* motif lies in a region where *RLG_Quinta* and *RLG_Cereba* LTRs are completely different (see Fig. 1), we assumed that *RLG_Quinta* has acquired this motif from another genomic source. Indeed, we found a homologous sequence inside the LTRs of the *Copia* retrotransposon family *RLC_Gisela* (Suppl Fig. 9C). We propose that *RLG_Quinta* acquired this motif, for example through gene conversion, thereby replacing the ancestral LTR segment.

## Conclusions

Our study provided detailed insight into the role of centromere-specific retrotransposons in the function and evolution of centromeres of Einkorn wheat (*T. monococcum*). The diploid *T. monococcum* has diverged from the A genome of hexaploid wheat less than 1 million years ago [28, 31], suggesting the situation to be very similar in the two. Additionally, previous studies found that parts of *RLG_Cereba* and *RLG_Quinta* retrotransposons have a strong propensity to associate with or phase CENH3 in the A, B and D subgenomes of hexaploid wheat [22, 27, 58]. Furthermore, genomic organization and TE content of all three wheat subgenomes are very similar [66, 69]. We therefore suggest that our findings also hold true for the three subgenomes of hexaploid wheat, despite them having diverged 3–7 million years ago [28, 31].

We found that the non-autonomous *RLG_Quinta* family evolved from autonomous *RLG_Cereba* retrotransposons at least 28 million years ago in the ancestor of Triticeae and oat [42]. We propose that *RLG_Quinta* relies on the RT and INT proteins of *RLG_Cereba*, but presumably also contributes GAG proteins which are needed in high numbers for the virus-like particles in which reverse transcription takes place. During their evolution, the LTRs of *RLG_Cereba* and *RLG_Quinta* evolved the function to phase and/or position CENH3-containing nucleosomes, rendering the ancestral centromere-specific tandem repeats unnecessary and leading to their eventual loss in the *T. monococcum* genome. Here, *RLG_Quinta* went through a particular evolutionary step when it acquired the novel *QuintCent* sequence motif from another retrotransposon. This gave it a strong propensity to precisely place nucleosomes in a manner that is strikingly similar to the strict placing of nucleosomes in the LTR of HIV [36]. Retroviruses are widely believed to have evolved from *Gypsy* retrotransposons in animals [41]. However, it is still intriguing that nucleosome phasing in their LTRs has remained so similar despite HIV and plant centromere-specific retrotransposons having diverged hundreds of millions of years ago.

We therefore suggest that *RLG_Quinta* has evolved beyond being purely a parasite of *RLG_Cereba*. On one hand it contributes to the replication process of both *RLG_Cereba* and *RLG_Quinta* through the contribution of GAG proteins. On the other hand it contributes to the function of the host centromere. We propose that, in this way, *RLG_Quinta* evolved from a purely parasitic to mutualistic genomic element.

**Fig. 7:**
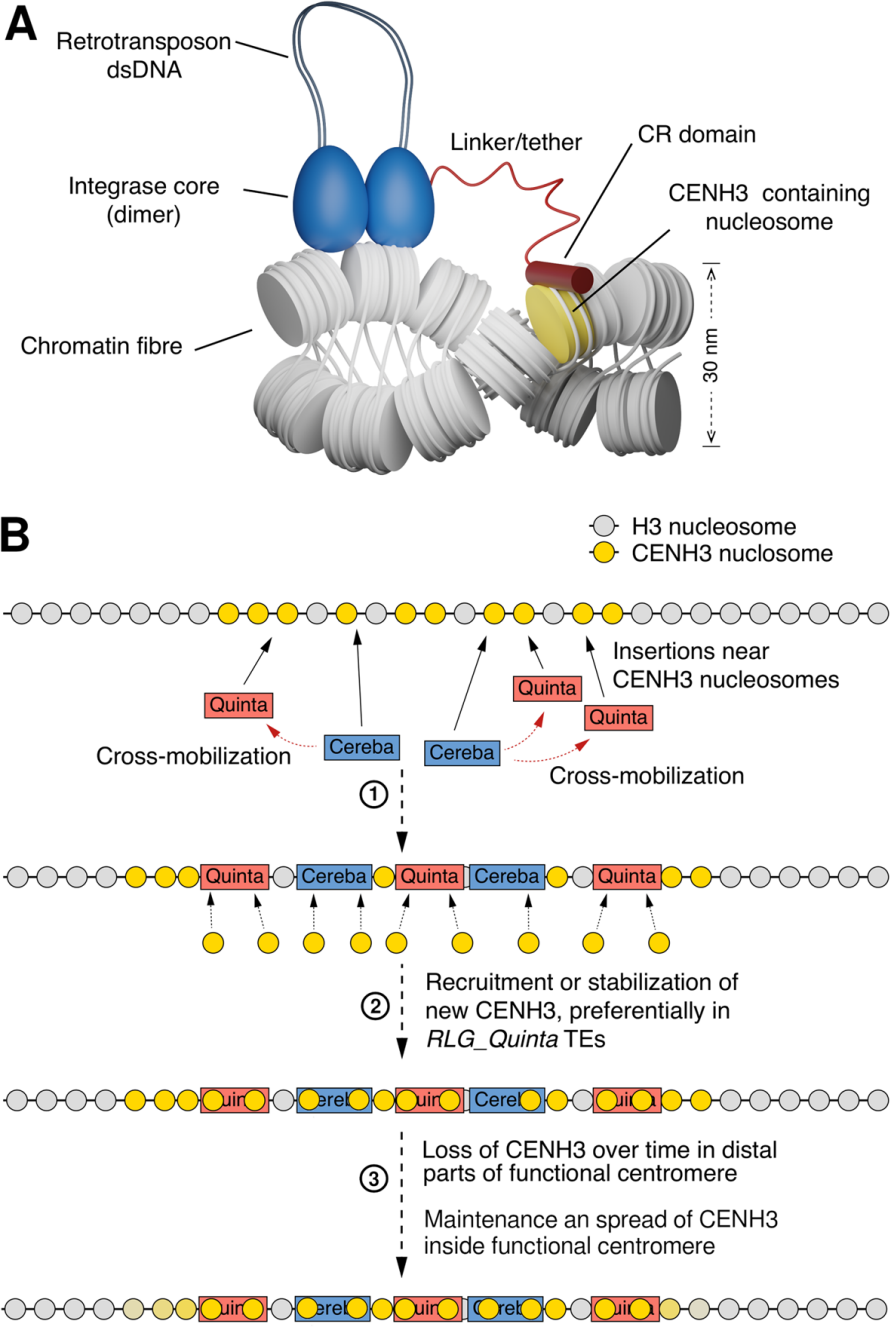
Models for the function and dynamics of retrotransposons in *T. monococcum* centromeres. **A** Schematic simplified model of the *RLG_Cereba* integrase complex. The CR domain specifically binds directly to CENH3 in nucleosomes, thereby ensuring selective insertion into functional centromeres. The CR domain is connected through a  tether/linker to the integrase core enzyme, directing the insertion of the double-stranded DNA (dsDNA) to a nearby loop in the chromatin fiber. **B** Sequence turnover and maintenance of centromeres. Step 1: The nucleosomes in and around centromeres are shown simplified by colored circles. The autonomous *RLG_Cereba* retrotransposons (blue boxes) replicate and insert copies of themselves into centromeres. Additionally, they also cross-mobilize *RLG_Quinta* retrotransposons. New *RLG_Cereba* and *RLG_Quinta* copies are inserted near nucleosomes that contain CENH3 histone variants. Step 2: The presence of *QuinCent* sequence motifs (see Fig. 6) in *RLG_Quinta* promotes recruitment of new CENH3 histone variants through unknown mechanisms. Step 3: Over time, CENH3 variants in distal parts are lost, thereby maintaining size the functional centromere. Additional CENH3 may be deposited in the functional centromere over time

We combined our findings in a model for the evolution and current dynamics of wheat centromeres (Fig. 7). Our data on insertion ages indicate that *RLG_Cereba* retrotransposons are consistently active, providing a steady flow of new insertions into active centromeres. *RLG_Cereba* retrotransposons also cross-mobilize *RLG_Quinta* retrotransposons (Fig. 7A, step 1). Here, the two retrotransposons presumably have complementary functions: first, *RLG_Cereba* contributes enzymes for replication and insertion, while *RLG_Quinta* provides GAG proteins in the required high quantities for the formation of virus-like particles in which replication takes place. Second, the CR domain attached to the *RLG_Cereba* INT enzyme ensures that new *RLG_Cereba* and *RLG_Quinta* copies are inserted into the functional centromere. Third, newly inserted copies then promote phasing/placement of CENH3-containing nucleosomes to their LTR sequences (Fig. 7A, step 2). Over time, the physical growth of the functional centromere through new retrotransposon insertions is compensated through loss of CENH3 in its distal regions (Fig. 7B, step 3). It is possible that centromere size is simply determined by the amount of available CENH3 protein [68]. Additional CENH3 may be accumulated in the functional centromere over time through subsequent epigenetic processes [32, 33].

The precise molecular mechanism of CENH3 deposition in plants is still an active research field, and our study can only contribute evidence that specific sequences (such as *QuinCent*) may be essential. Indeed, previous work found a strong association of *RLG_Quinta* sequences with CENH3 deposition [22]. We also emphasize that we only provide bioinformatical evidence for the interaction of the CR domain with CENH3. It was beyond the scope and resources of this study to provide functional proof, and extensive wet lab experiments such as Co-immuno precipitation will be needed to conclusively demonstrate a direct protein–protein interaction. Despite its limitations, our study highlights the complex interplay of a pair of autonomous and non-autonomous retrotransposons in the environment of plant centromeres. Whether this quasi-symbiotic relationship is an exception that only arose in Triticeae or whether mutualistic pairs of retrotransposons shape the centromeres of other plants remains an open question.

## Methods

### Software sources

Unless stated otherwise in the methods section, bioinformatics software was obtained from Ubuntu repositories (ubuntu.com).

### Transposable element annotation

Full-length *RLG_Cereba* and *RLG_Quinta* retrotransposons were identified and annotated with the previously described TEpop pipeline [71] which uses multiple consensus sequences of LTRs and searches for occurrences of LTRs in the same orientation and in the distance from each other that is roughly expected from the length of the consensus sequence of the respective retrotransposon family. In a second step, candidate full-length copies are screened for the presence of the predicted coding sequences (CDS) to discard cases in which two LTRs were found at the right distance by chance. For *RLG_Cereba* retrotransposons, we selected copies ranging in size from 7,700–8,000 bp, to also allow for copies that contain small insertions or deletion. *The RLG_Quinta* populations are more complex, as they contain two groups that differ in size due to variable lengths of the predicted UTR. Here, we size-selected for copies of 4,300–4,500 bp and 4,700–4,800 bp, respectively. Full-length *RLG_Cereba* and *RLG_Quinta* retrotransposon homologs were isolated from the *T. monococcum* TA299 and T10622 genome assemblies [2], as well as from the previously published genome assemblies of barley [29], *Brachypodium* [65], oat [20], rye [47], maize [17], rice [21], *Pharus latifolius* [26] and *Streptochaeta angustifolia* [52].

### Retrotransposon insertion age estimates

Insertion ages of individual retrotransposon copies were estimated by aligning the two LTRs of each copy with the EMBOSS program Water (obtained from Ubuntu repositories, ubuntu.com), using a gap opening penalty of 10 and a gap extension penalty of 0.5. Nucleotide differences between LTRs were counted and transitions and transversions were distinguished for molecular dating as previously described [5]. For all molecular dating, a rate of 1.3E-8 per site per year proposed for intergenic regions in grasses was used [25]. Molecular dating of insertions times was automated with the in-house Perl script date_pair.

### Analyses of populations of *RLG_Cereba* and *RLG_Quinta* retrotransposons

All identified full-length *RLG_Cereba* and *RLG_Quinta* retrotransposon copies were aligned to their respective consensus sequences using the EMBOSS program Water, using a gap opening penalty of 50 and a gap extension penalty of 0.1. The alignments were then transformed into a variant call file (vcf) with the previously described Perl script pair_to_vcf [71]. Sequence variants were used if they occur in at least 1% of all retrotransposon copies (i.e. minor allele frequency of 1%). Insertions in retrotransposon copies were ignored, and deletions were treated as missing data, with a missing data cutoff at 90%. The vcf file was then used for principal component analysis (PCAs) using the R libraries gdsfmt, SNPRelate, ggplot2 and magrittr. Consensus sequences for defined subfamilies were constructed from 30 randomly picked full-length copies, which were aligned with Clustalw at default settings.

The consensus sequences for individual subfamilies were then used for subsequent analyses which included: (i) prediction of hypothetical encoded proteins, (ii) comparisons between *RLG_Cereba* and *RLG_Quinta* retrotransposons (shown in Fig. 1a), and (iii) multiple alignments of LTRs and PBS and PPT regions (shown in Fig. 1B and C). Hypothetical proteins were used for sequence comparisons and phylogenetic analyses (Fig. 2A, Supplementary Fig. S9).

### Analysis of CENH3 ChIP-seq data

Mapped reads for CENH3, H3K4me2 and their respective controls were downloaded from: (10.5061/dryad.0p2ngf24b). The samtools depth command was used to calculate per base sequence coverage. The CENH3 read depth coverage was then cross-matched with our TE annotations. For the calculation of average CENH3 read coverage per TE-copy, all annotated TEs of at least 1000 bp were used. For the identification of specific TE regions with high CENH3 read coverage, the individual annotated copies were aligned to the consensus sequence of the respective TE family, omitting insertions in the aligned copies in order to map CENH3 hot spots on the TE consensus sequence.

For the identification of CENH3-specific hot spots, the ChIP-seq read mappings were screened for segments of at least 100 bp where CENH3 read coverage was at least 5 times the average CENH3 coverage, and at least 5 times higher than the sum of the read coverage of all control and mock experiments. As control, we also searched the terminal 100 Mb of chromosome 1A for such peaks. Because of high enrichment of CENH3 in centromeres, the average CENH3 coverage was calculated once only for all predicted centromeric regions and for the control for the data from the terminal 100 Mb of chromosome 1A. The same procedure was used for C1_H3K4me3 ChIP-seq data. The sequence peaks identified in this way where then used in blastn searches against the TREP database (www.botinst.uzh.ch/en/research/genetics/thomasWicker/trep-db.html). Segments were classified as belonging to a given TE family, if they produced blastn alignments >= 90 bp and had at least 70% sequence identity.

### 3D protein modelling and visualization

3 dimensional protein models were generated using ColabFold/Alphafold2 (10.1038/s41592-022-01488-1 / 10.1038/s41586-021-03819-2), using the following settings: template_mode = none, mas_mode:MMseqs2(UniRef + Environmental), num_recycles = 3. Protein structures were visualized usingPyMOL(https://pymol.org). The schematic model for the complex of *RLG_Cereba* integrase with the chromatin fiber (shown in Fig. 7B) was created using Blender (http://www.blender.org).

### Identification of *RLG_Cereba* and *RLG_Quinta* homologs in species other than wheat

The predicted protein sequences containing the GAG protein from *T. monococcum RLG_Cereba* and *RLG_Quinta* consensus proteins were used in tblastn searches against the genomes of Brachypodium, barley, oat and rice. All regions producing blast hits > 90 aa and > 35% protein sequence identity were extracted from the respective genomes adding 5000 bp of flanking sequence. The extracted sequences were then aligned using Clustalw. The alignments were then trimmed, and a consensus sequence was inferred using the in-house script visual_clustal. The consensus sequences were then visually inspected by dotplot e.g. for completeness of LTRs.

### Consensus sequences of CR domain containing retrotransposons

CR domains of retrotransposons were downloaded from [38]. The CR domains were taken as a seed for blastn queries against their respective genome of origin [3, 7, 13, 16, 17, 19, 21, 23, 34, 40, 48, 49, 56, 62, 67, 72, 76–78] regions producing blast hits were extracted and processed as described above to generate consensus sequences.

### Phylogenetic analyses of consensus sequences

DNA or protein sequences were aligned with CustalW. Multiple alignments were converted to nexus format with Clustalx. Alignments were visually inspected for proper alignment of sequences. Phylogenetic trees were constructed with MrBayes using the mcmc algorithm. Generations were added until the average standard deviation of split frequencies dropped below 0.01. For trees of nucleotide sequences, the option lset nst = 6 rates = invgamma was used. For all trees, a burn-in of 25% was used. Phylogenetic trees were visualized with FigTree.

### Phylogenetic analyses of individual TE copies of Einkorn

To exclude recombined elements we first filtered the isolated TE copies, based on the similarity between the 5’ and 3’ target site duplication, allowing for 1 mismatch. The copies passing this filtering were then aligned with MAFFT version 7.525 with the following parameters: –reorder –maxiterate 1000 –nomemsave –leavegappyregion –6merpair. Maximum likelihood trees were then estimated with RAxML version 8.2.12 as described in (10.1371/journal.ppat.1011130). Briefly, 10 maximum likelihood trees were estimated with: raxmlHPC-PTHREADS-SSE3 -m GTRGAMMA -p 12,345 -# 10 –print-identical-sequences -s [alignment].phy. Then we performed boostrap analysis on the best tree using the parameters: raxmlHPC-PTHREADS-SSE3 -m GTRGAMMA -p 12,345 -b 12,345 -# 50 –print-identical-sequences -s [alignment].phy -t [best_tree] and the bipartition were added to the best tree with: raxmlHPC-PTHREADS-SSE3 -m GTRGAMMA -p 12,345 -f b –print-identical-sequences -s [alignment].phy -t [best_tree_with_bootstrap]. Information on whether an individual copy is part of a centromere was obtained from [2]. To determine whether a copy likely inserted into the newly formed portion of the Chr4Am centromere, copies had to be located in the genomic region of 281.1 Mb-284 Mb and have an estimated insertion ago of 100′000 years or younger, as the centromere shift was dated to approximately 30′000–100′000 years ago. Phylogenetic trees with the corresponding annotations were visualized in R using the packages: ggplot2, gtree, ggtreeExtra and ggnewscale.

### Mantel test for association between genetic distance and genomic position of TEs

The vcf file obtained from TEpop was converted to hapmap and then reformatted using TASSEL5 and R. Pairwise comparison of all individual elements to count SNPs was done using the dist.gene function from the ape package. The difference of genomic positions was calculated in relation to the midpoint of the centromere of the respective chromosome where a TE copy is situated. This means that if the centromere midpoint of a chromosome is at 250 Mb and a TE copy is at 245 Mb the genomic position in relation to the centromere midpoint was counted as 5 Mb. Another copy situated at 255 Mb would also be counted as 5 Mb meaning that they have no positional difference for this analysis. Association between positional and genetic distance was then assessed using the R function mantel() with the parameters: method = ”spearman”, permutations = 9999, na.rm = TRUE. Linear regression was calculated using the R function lm() with the formula x ~ y.

### Supplementary Information

Supplementary Material 1.

## Data Availability

Source data not otherwise publicly available can be accessed at: https://github.com/Wicker-Lab/wheat_centromere_materials.
