# Supplementary material for: Evolution of Einkorn wheat centromeres is driven by the mutualistic interplay of two LTR retrotransposons
Source: Mob DNA. 2024 Aug 5;15:16. doi: 10.1186/s13100-024-00326-9 (PMC11302176; doi:10.1186/s13100-024-00326-9)
Supplement: Supplementary file 1 — Supplementary Material 1. [file 13100_2024_326_MOESM1_ESM.pdf]

## Supplementary Figures

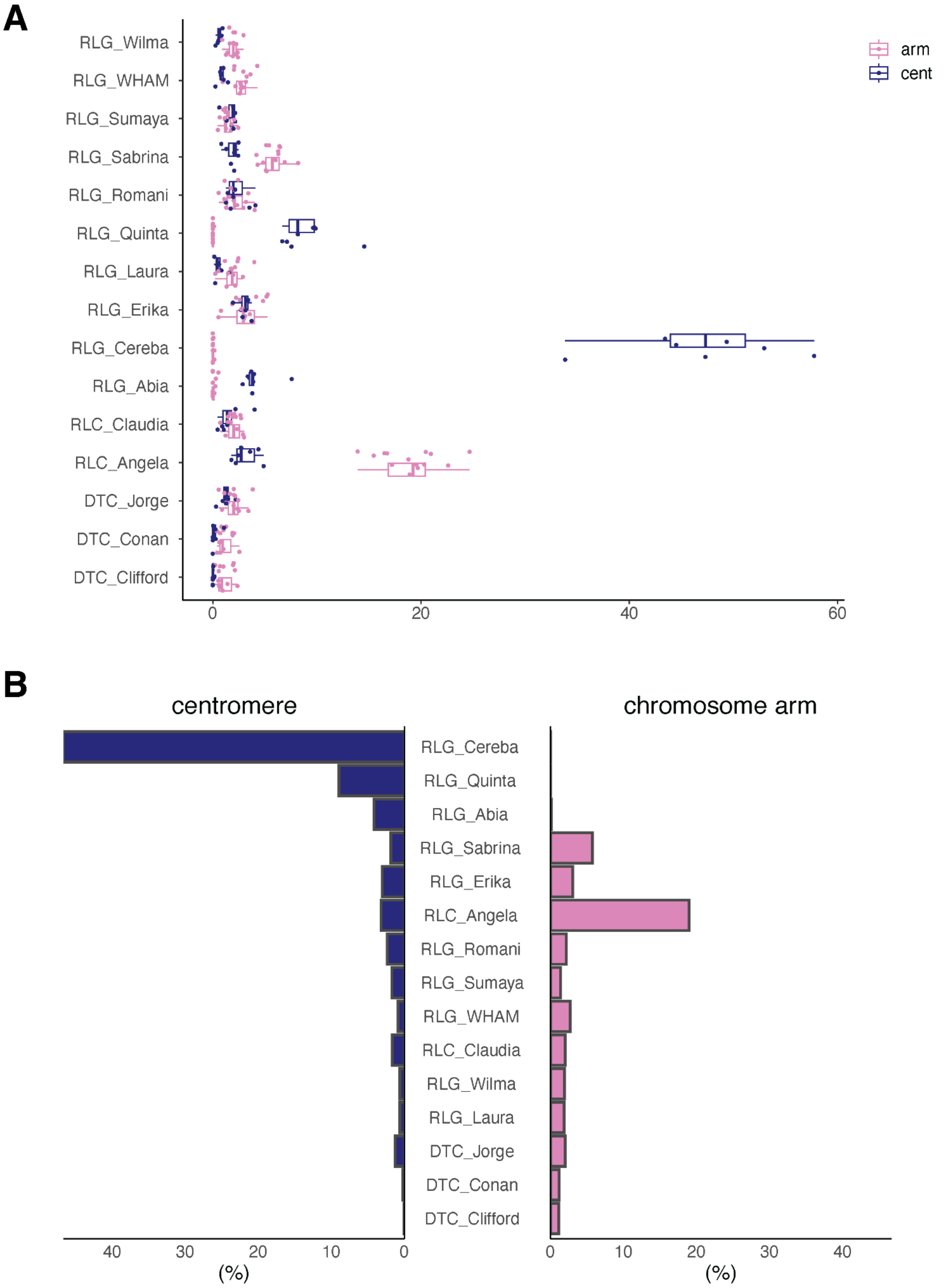


**Supplementary Fig. S1**. Relative abundance of the 15 most abundant TE families in centromeres and in distal chromosome parts in *T. monococcum*. A. Contribution of TE families (in %) to centromeres (blue) and distal regions (pink). For distal regions, two segments (position 50-55Mb and 60-65 Mb) from the short arm of all chromosomes were used. Each dot represents TE abundance in one individual chromosomal segment. B. Cumulative contributions of TE families to all centromeres (blue) and all distal chromosomal segments.

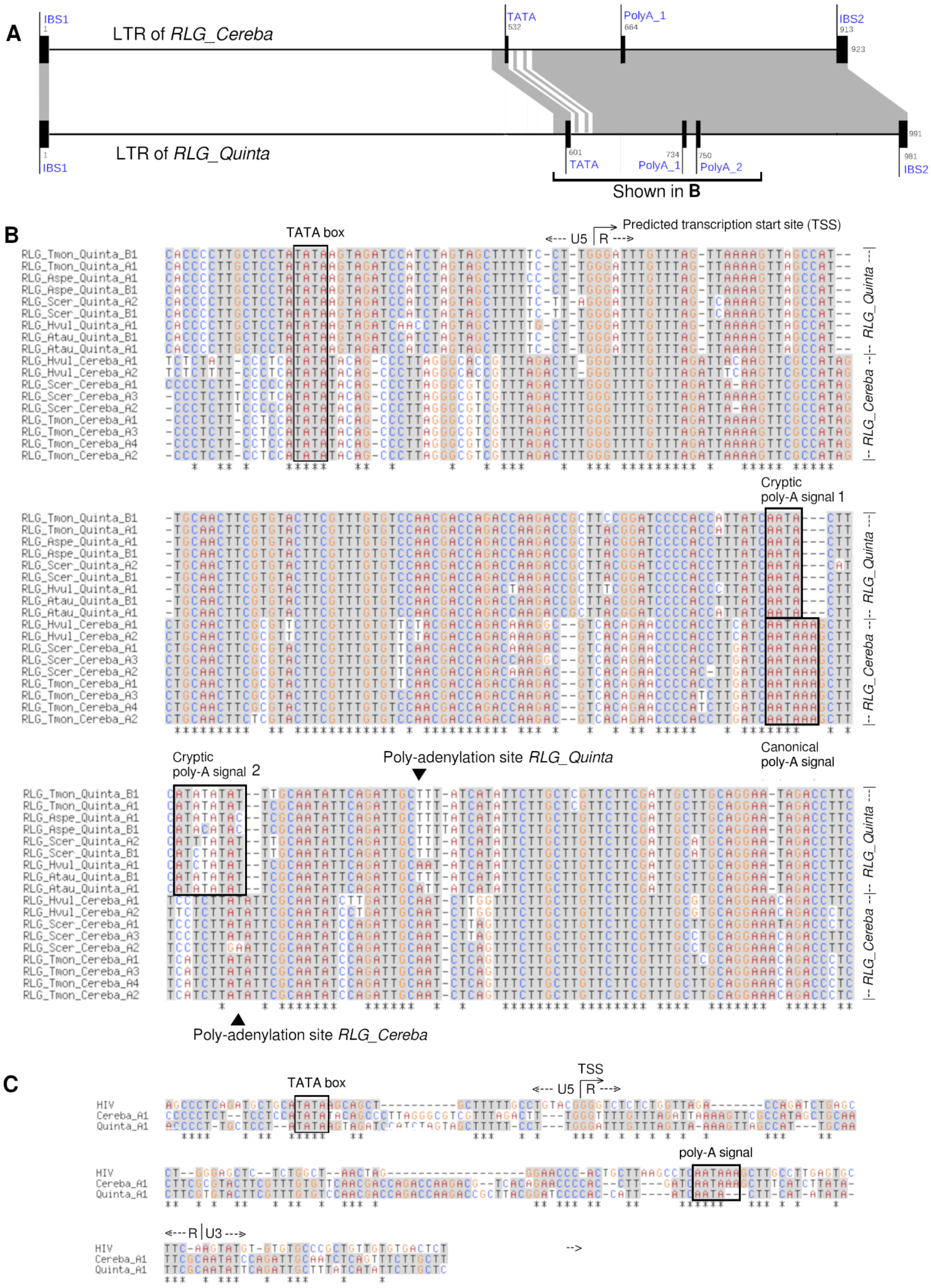


**Supplementary Fig. S2.** Putative functional motifs in LTRs of *RLG_Cereba* and *RLG_Quinta*. **A.** Schematic comparison of LTRs from *RLG_Cereba* and *RLG_Quinta*. Conserved stretches of sequences are indicated by gray areas. IBS: predicted integrase binding site. **B.** Partial multiple alignment of LTRs from *RLG_Cereba* and *RLG_Quinta* subfamilies. Poly-Adenylation sites were predicted from IsoSeq transcripts, and the transcription start site from homology with Human Immunodeficiency Virus (HIV). **C.** Comparison of conserved putative regulatory elements in LTRs from *RLG_Cereba, RLG_Quinta* and Human HIV (Genbank accession AY352275).

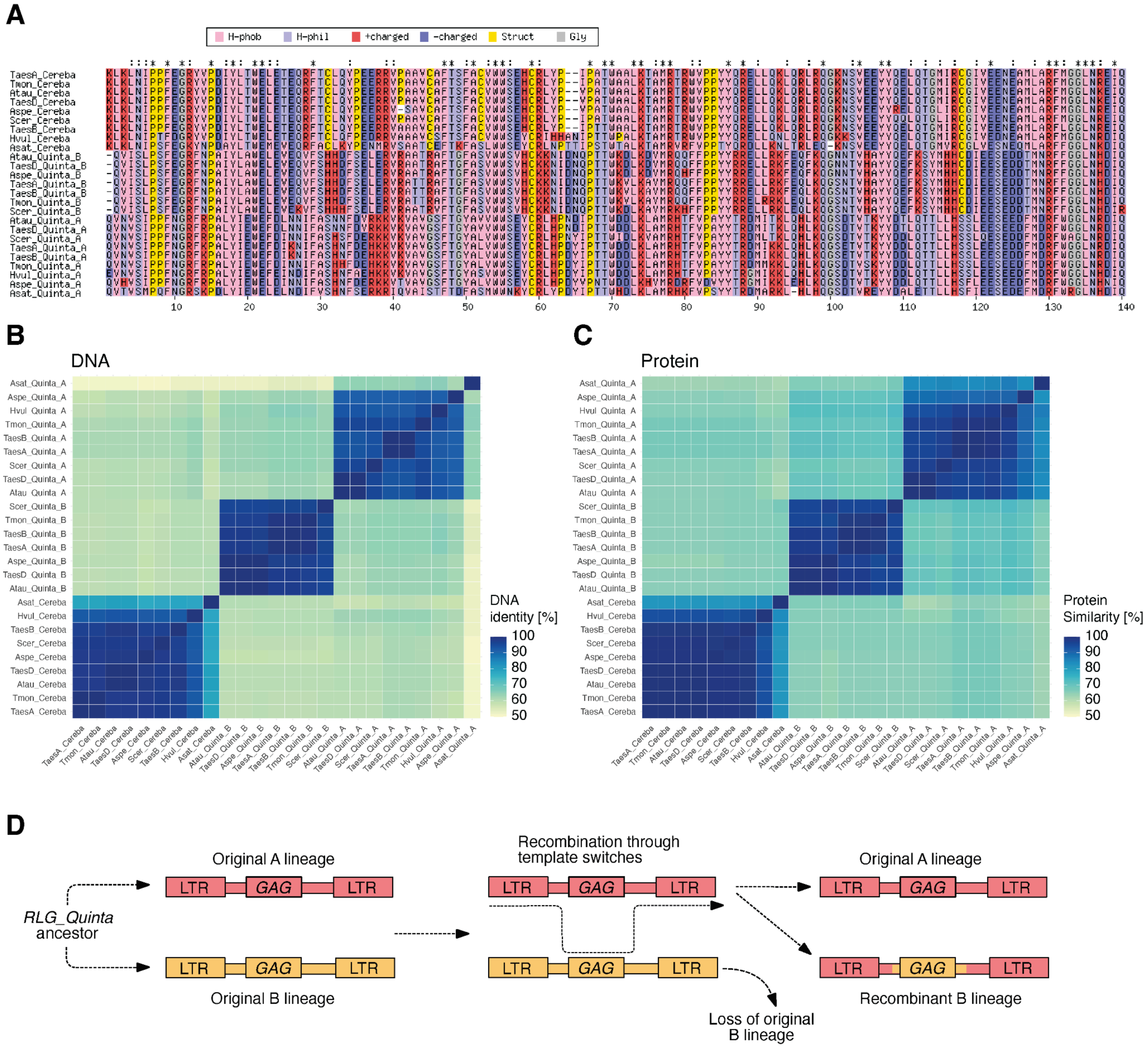


**Supplementary Fig. S3**. Evolution of the *RLG_Cereba* and *RLG_Quinta* GAG proteins. **A.** Multiple alignment of predicted GAG proteins that was used for the construction of the phylogenetic tree in Fig. 2. Note that sequences from *RLG_Cereba*, *RLG_Quinta_A* and *RLG_Quinta_B* form three distinct groups, indicating they diverged early on, in the ancestor of Triticae and *Avena sativa*. Species abbreviations are the same as in Fig. 2A. **B.** Heat map of DNA sequence identities of GAG coding sequences (CDS). Note that sequence identity between the three main groups (*RLG_Cereba*, *RLG_Quinta_A* and *RLG_Quinta_B*) is very low (50-60 %). DNA alignments could only be done based on a codon-by-codon alignment derived from the multiple alignment of the proteins shown in (A). **C.** Heat map of protein similarities of GAG proteins. **D.** Schematic and simplified model that explains why the GAG gene is highly divergent between the *RLG_Quinta* A and B lineages. We propose that the A and B lineages diverged early in the *RLG_Quinta* evolution. At some point (e.g. through template switching during TE replication), the CDS of the B lineage was recombined with the up- and downstream sequences of the A lineage, while the original B lineage went extinct.

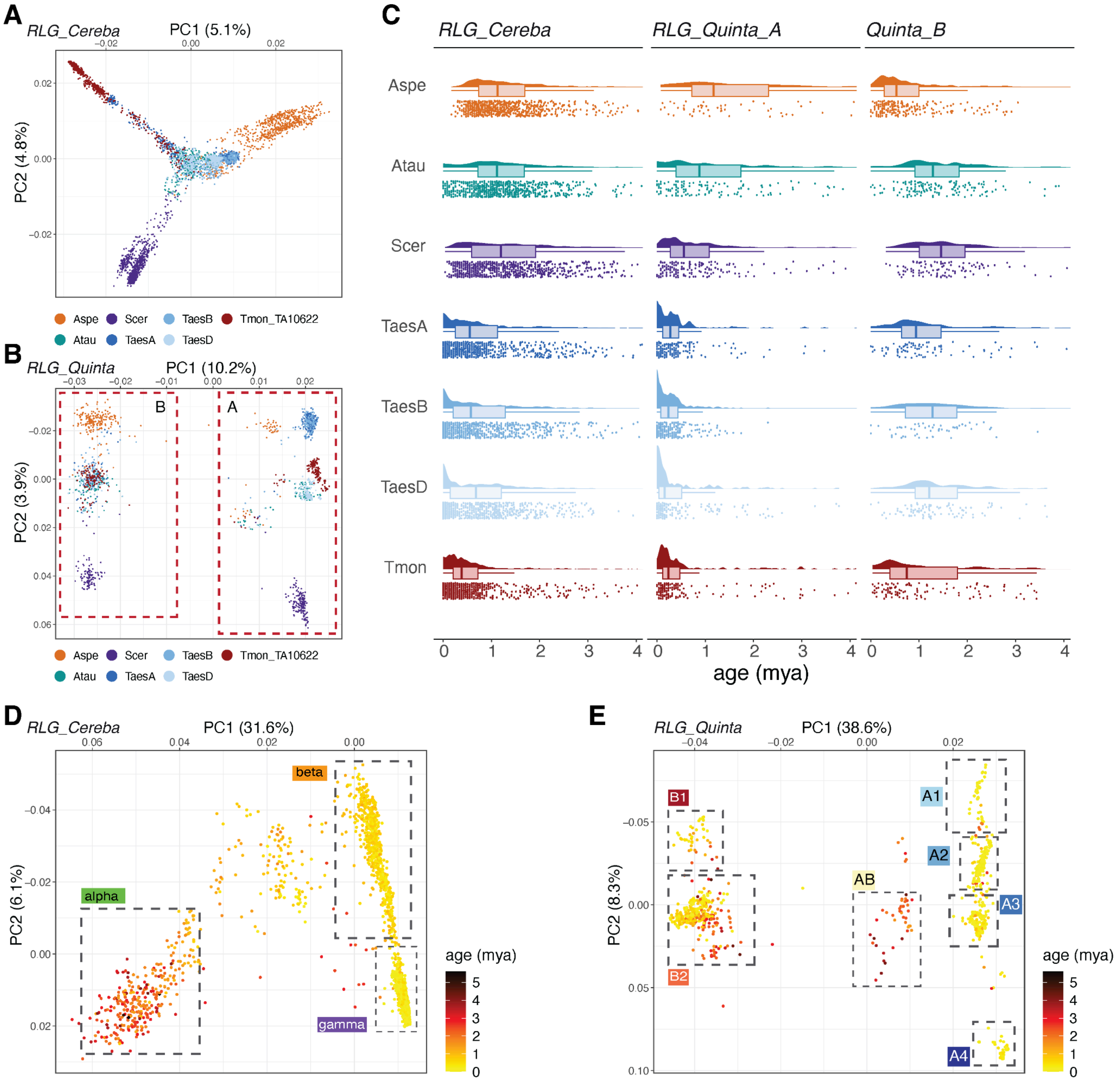


**Supplementary Fig. S4.** Analysis of populations of centromere-specific retrotransposons. **A.** Principal component analysis (PCA) of full length *RLG_Cereba* elements using SNPs obtained from alignment of individual elements against a consensus sequence. For each species, 747 randomly picked elements were included in the analysis. Retrotransposons form separate groups which largely correspond to the species/subgenomes used (Aspe: *Ae. speltoides*, Atau: *Ae. tauschii*, Scer: *S. cereale*, Tmon: *T. monococcum* accession TA10622, Taes: *T. aestivum*, A, B and D subgenomes). **B.** same analysis as in (A) but using 274 randomly picked *RLG_Quinta* elements from each species/subgenome. A and B clusters include elements from all the included species. **C.** Insertion age distributions estimated from LTR divergence of *RLG_Cereba* and *RLG_Quinta* elements. Colors correspond to those of the subfamilies shown in the PCA in A and B. For visual clarity only data points falling into the 99% percentile are shown. The most recently active *RLG_Quinta* elements in the *T. aestivum* subgenomes and *T. monococcum* are part of the B lineage, while the A lineage was more recently active in the genome of *Ae. speltoides*. **D.** Principal component analysis (PCA) of only Einkorn (accession TA10622) *RLG_Cereba* elements using SNPs obtained from alignment of individual copies against a consensus sequence. **E.** The same analysis with *RLG_Quinta* copies.

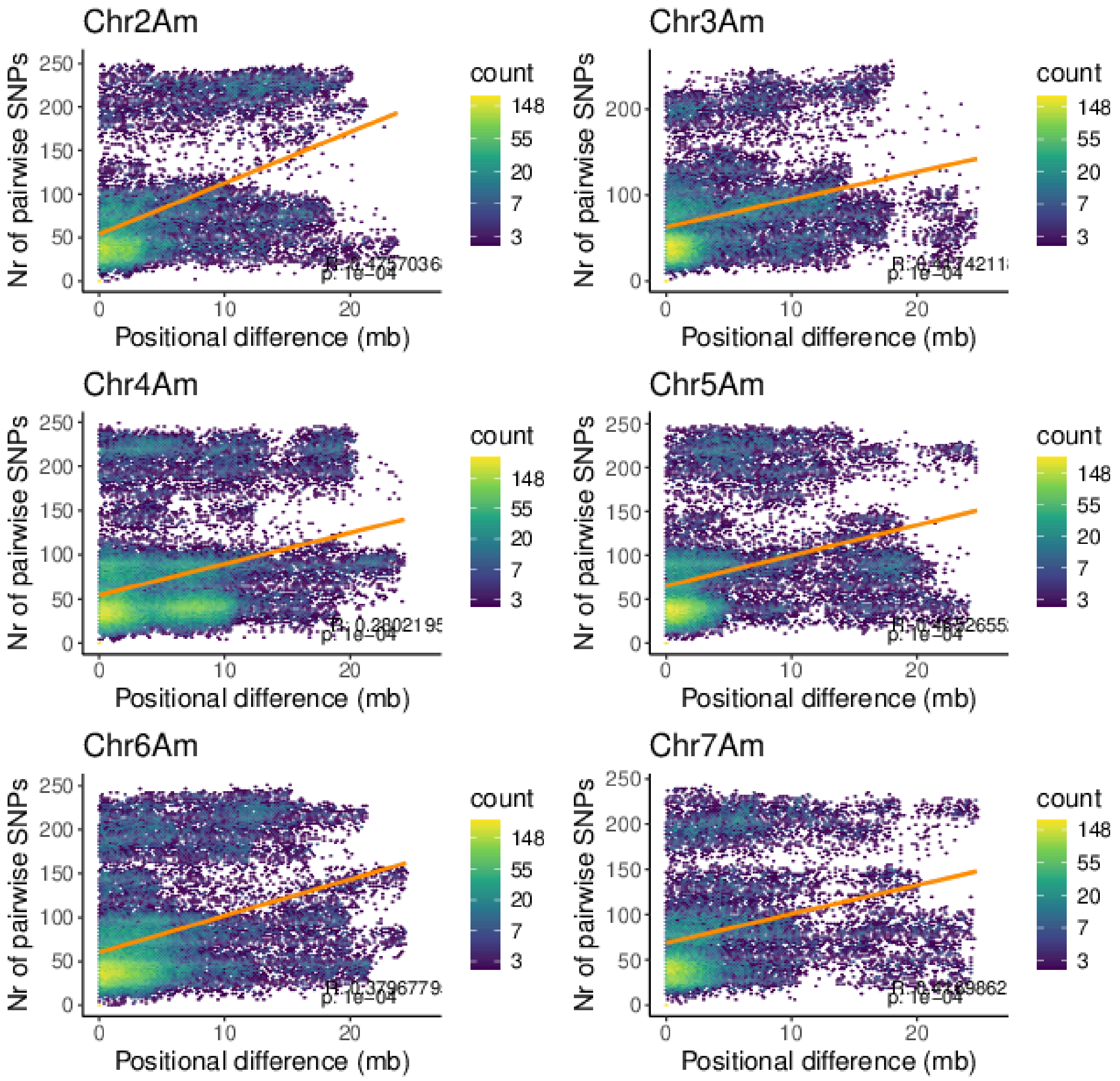


**Supplementary Fig. S5.** Association of physical to genetic distance of *RLG_Cereba* retrotransposon copies in centromeres of *T. monococcum*. The x-axis indicates the difference in genomic position, measured by the absolute difference in the distance from the centromere midpoint. The y-axis shows the number of SNPs in pairwise alignments of individual copies. Mantel test statistics and the corresponding p-value are shown in the bottom right of each plot. The orange line shows the linear regression calculated by the function lm().

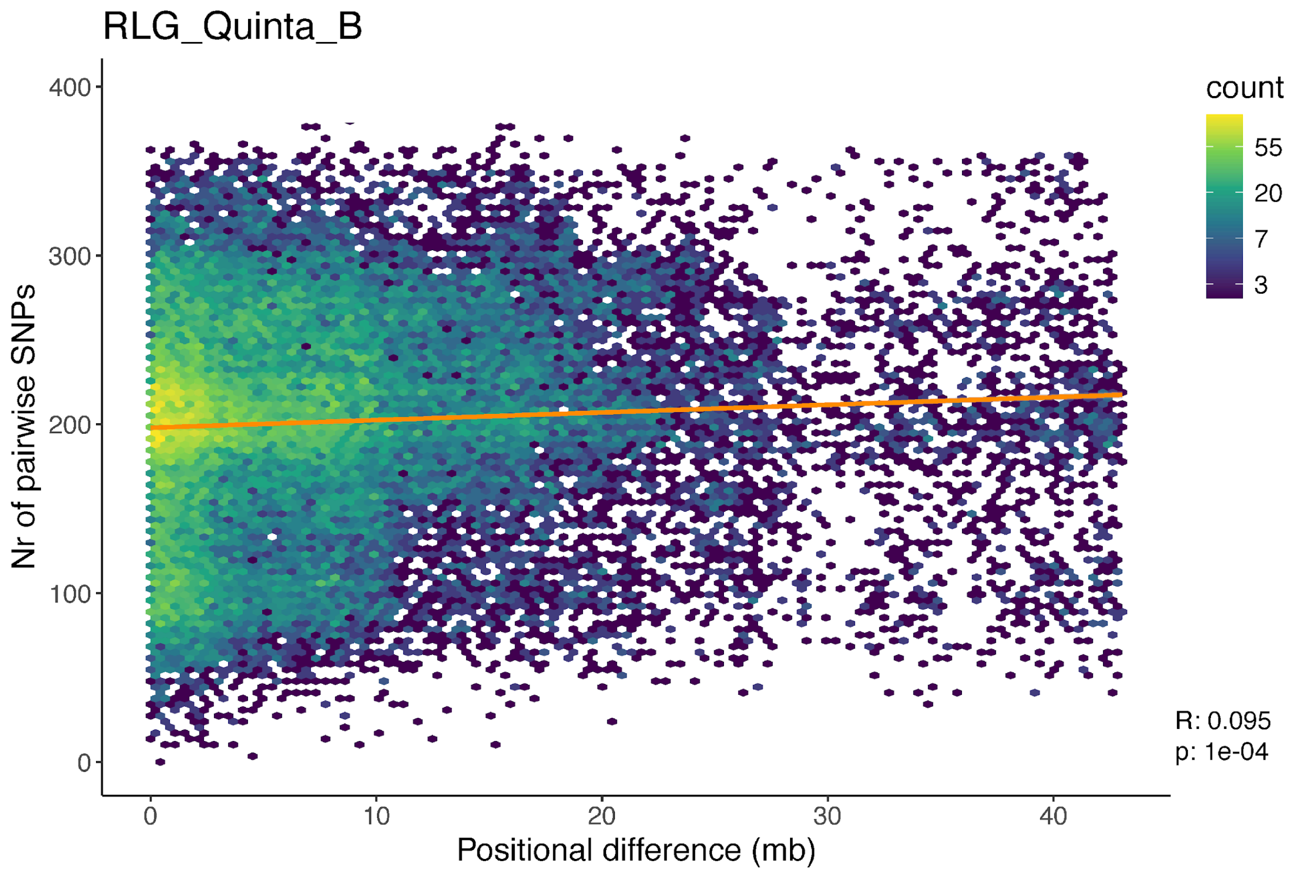


**Supplementary Fig. S6.** Association of physical to genetic distance of *RLG_Quinta_B* retrotransposon copies in centromeres of *T. monococcum*. The x-axis indicates the difference in genomic position, measured by the absolute difference in the distance from the centromere midpoint. The y-axis shows the number of SNPs in pairwise alignments of individual copies. Mantel test statistics and the corresponding p-value are shown in the bottom right. The orange line shows the linear regression calculated by the function lm().

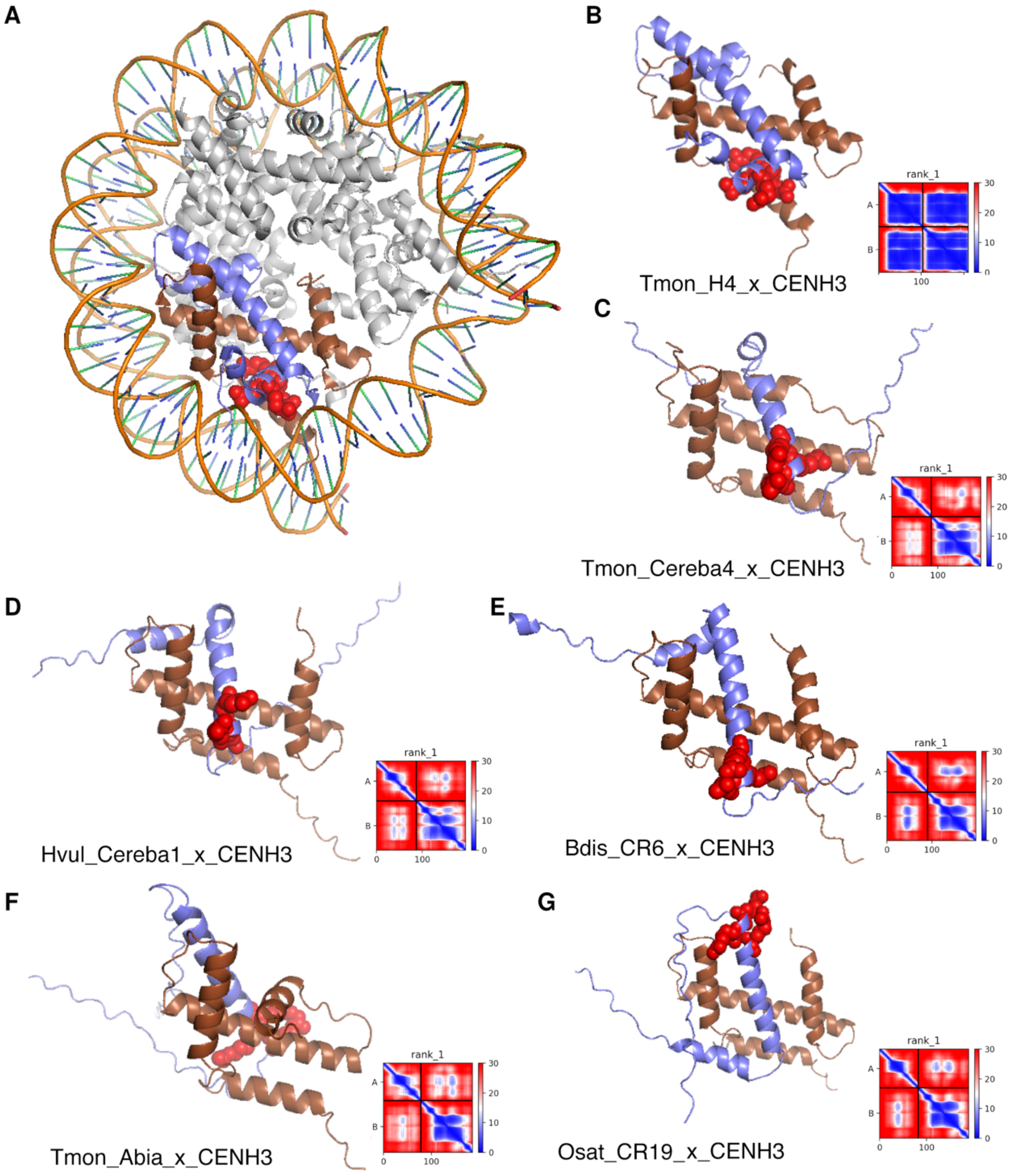


**Supplementary Fig. S7.** Three-dimensional models for interactions of CENH3 with histone H4 and CR domains from centromere-specific retrotransposons. CENH3 is shown in brown and H4 and CR domains in light blue **a.** Crystal structure of human nucleosome (Tachiwana et al. 2011) with one CENH3 (CENP-A) und one H4 unit in color. **b.** Alphafold2 multimer model of interaction of CENH3 and H4 from *T. monococcum*. There, a long H4 helix lies across a groove in CENH3 and interacts on its other side with the DNA backbone via a series of positively charged amino acids (RRxRR motif). **c.** through **g.** Alphafold2 multimer models of interactions of representative CR domains with *T. monococcum* CENH3. Positively charged amino acids of the CR core motif (RxRxR/K) which are in a similar spatial location as the H4 RRxRR motif are shown as red spheres. Only the best ranked model is shown, along with the corresponding predicted aligned error (PAE) plot. In the PAE plot, sequence A corresponds to the CR domain while sequence B corresponds to CENH3. The CR domain seems unorganized except for two alpha-helices that are predicted to interact with CENH3 showing high PAE values. Interestingly, 6 out of 11 CR domain models (example in **c.** through **e.**) show a very similar binding of CR to CENH3, with the RxRxR/K motif being in the same place as the residues that interact with the DNA back bone in histone H4. **f.** and **g.** are representatives of alternative interaction found in predictions for 5 of the CR domains.

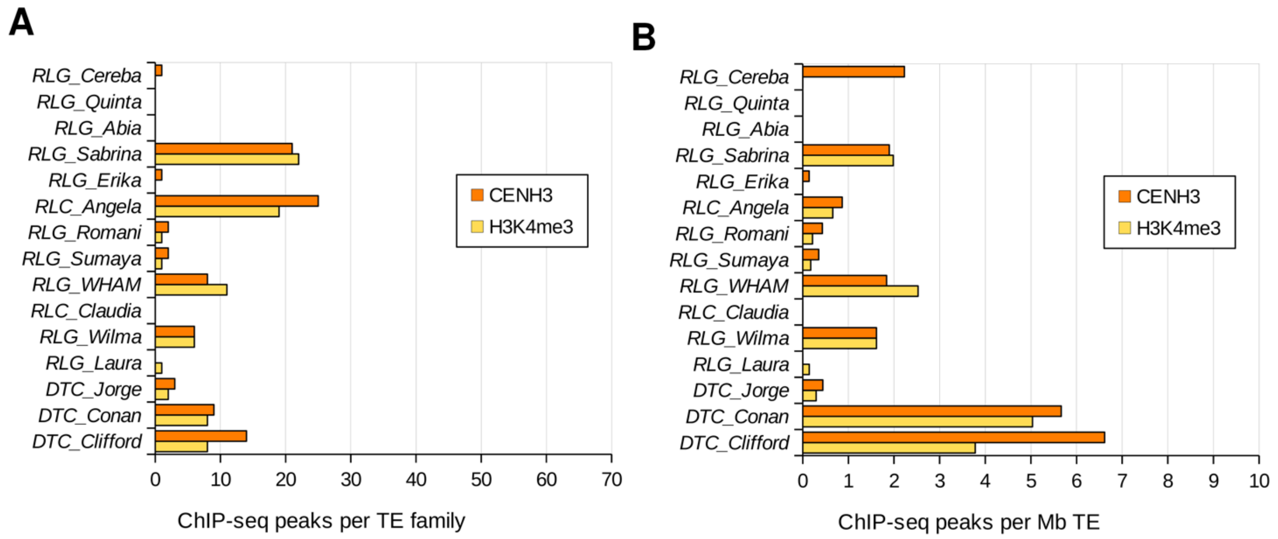


**Supplementary Fig. S8**. Distribution of sequences associated with CENH3 and H3K4me3 among high-copy TE families. **A.** Numbers of CENH3 and H3K4me3 peaks found in TE families in the terminal 20 Mb of chromosome 1A of *T. monococcum*. **B.** relative abundance of CENH3 and H3K4me3 peaks per Mb for each TE family. Overall, CENH3 and H3K4me3 peaks are much less numerous in TE families in the distal part of chromosome 1A than in centromeres (see text). Additionally, the numbers of CENH3 and H3K4me3 peaks differ only little.

**A**

```
>RLG_Tmon_Quinta_A1_consensus-1_LTR
TGATGAGGACATGACTACCTTGGATACGACCAAAAATATTGCATACATGC 50
ATATTTGTCAGGTGATTTCTAATGCAAACTATTCAATAATCTATTTATGT 100
TATCCAGAACAAAAATACTTCACACACTTTTGTGTTTTGTCTAATTGCAG 150
GTGTATGGGACATTTGTACAATCCACATATGAAGATAAGGGAAAAGGAGA 200
AGTTTAGGTTGTGTTCAAAAAGTCCTCTCACGTCACTTTTGGGCCAAGAG 250
AAGATAGAGTCCAAGTCTCTCACGTTCTGGATTCAGATTCGGACTGCACA 300
GACATACCTGACTCAAAACGCCAACAACTTTTTCATACGGACTCCGAATT 350 U5
GGGTGATTCTTTTTTTGTTGGAAACTAGATTTCGTGCTCTTTCCAACCCA 400
ATTGGATTCACCTTCAAATTCGTCCGGAGCGTTGAGTTATGGACGAAACA 450
ATCTGACGTTGCAGCAGAATCCGAGTCAAACTACAAGTCCAAAGGTGTTG 500
CATCACCTCCACTTGGGCCCATGAGCCTTGTACGACCTAGGGTTAGTTTT 550
AGGCTGCCTTGGGACGTCCTCCCACCTCCTTGGCCGCCACCCCTTGCTCC 600
TATATAAGTAGATCCATCTAGTAGCTTTTTCCTTGGGATTTGTTTAGTTA 650
AAAGTTAGCCATTGCAACTTCGTGTACTTCGTTTGTGTCCAACGACCAGA 700 R
CCAAGACCGCTTACGGATCCCCACCATTATCAATACTTCATATATATTCG 750
CAATATTCAGATTGCTTTATCATATTCTTGCTCGTTCTTCGATTGCTTGC 800
AGGAATAGACCTTCGTGGTCAGGCTGACCGTGCTTCCGGCATCGTCAGTA 850
ACCTCAGGAGATTGGTTTAGCGATTGCTAAGGCGCAACGTCGTGCACGTT 900 U3
TGTAGTCGGATCGTCAAAGTCGTCTCCACCAAATCGATAGTTATCATCTC 950
ATCGAAAGATCGGGACCCTCGCCTCTATCA 980
```

**B**

```
                    ---- - --->      - ---->
CAGACATACCTGACTCAAAACGTCAACAACTTTTTCATACGGACTCCGAA  50

   ----- ->   ->  -------->              <--------
TTGGGTGATTCTTTTTTTGTTGGAAACTAGATTTCGTGCTCTTTCCAACC 100

 <-   <- -----         <---- -      <--- - ----
CAATTGGACTCACCTTCAAATTCGTCCGGAGCGTTGAGTTATGGACGAAA 150

CAATCTGACGTT 162
```

**C**

```
RLC_Tmon_Gisela    -----TTTTCAGAACACCAAAACTGCCACAACTTTTGCAAACGGACTCCGAATTCGATGA
RLC_Hvul_Gisela    ---CTACCGCTGGATACTGAAACTGCTGTAACTTTCGCATACGGACTCCGAATTCAACGA
RLC_Asat_Gisela    CAAAAACTTCAGA--ACCAGAACTGCCACAACTTTTGCATCCGAACTCCAAACGAGATGA
RLG_Quinta_A1      CAGACATACCTGA--CTCAGAACGTCAACAACTTTTTCATACGGACTCCGAATTGGGTGA
                            * *        ***  *   ******  **  ** ***** **      **

RLC_Tmon_Gisela    AACCAAGTTTGTTGGAAAGCTAGCGACAAGGGCTAACACAATCTTGAAAGAAATATCAAT
RLC_Hvul_Gisela    AACCAAGTTTGTTGGAAAGCTAACGACATGGGCTAACACAATCTTGATAGAAATATCAAT
RLC_Asat_Gisela    AACCAATTTTGTTGGAAAGATAACGACGTG-------TAGAATCC--AATAAAATATTGGA
RLG_Quinta_A1      TTCTTTTTTTGTTGGAAACTAGATTTCGTG-------CTCTTTCC--AACCCAATTGGACT
                     *    ***********        *  *           **   *    ***

RLC_Tmon_Gisela    AAGAAGCAAATGAGAAAAGGCCCATAATAAAATGGTGAGAACCCTTCCTCGGA----
RLC_Hvul_Gisela    AAGAAGCAAGTGAGAAAAGTCCCATAAGAAAATGGTGAGAACCCTTCTTCGAATAAG
RLC_Asat_Gisela    TATCACCAAGGGAGAAAATACCATGAATATATATGTGAGATACCTCCCAAGGT----
RLG_Quinta_A1      CACCTTCAAATTCGTCCGGAGCGTTGAGTTATGGACGA-AACAATCTGACGTT----
                    *    ***    *       *    *   *     ** *    *     *
```

**Supplementary Fig. S9**. Analysis of a 162 bp sequence inside the LTR of *RLG_Quinta* which is strongly associated with placement of nucleosomes. **A.** Consensus sequence of *RLG_Quinta* LTR. In color are the predicted U5, R and U3 regions. The 162 bp motif is printed in bold and so are the predicted TATA box and poly-adenylation signals. B. The 162 bp sequence has a cryptic inverted repeat structure. Arrows above the sequence indicate complementary sequences. The 162 bp motif was possibly captured from another TE. The *RLC_Gisela* retrotransposons from *T. monococcum* (Tmon), barley (*H. vulgare*, Hvul) and oat (*A.sativa*, Asat) all have homologous sequences in their LTRs. The full-length *RLC_Gisela* sequences were deposited in the TREP database (www.botinst.uzh.ch/en/research/genetics/thomasWicker/trep-db.html).
